# Identification of a New FtsZ Inhibitor by Virtual Screening, Mechanistic Insights, and Structure–Activity Relationship Analyses

**DOI:** 10.1021/acsinfecdis.4c01045

**Published:** 2025-03-18

**Authors:** Pietro Sciò, Viola Camilla Scoffone, Anastasia Parisi, Marianna Bufano, Martina Caneva, Gabriele Trespidi, Samuele Irudal, Giulia Barbieri, Lisa Cariani, Beatrice Silvia Orena, Valeria Daccò, Francesco Imperi, Silvia Buroni, Antonio Coluccia

**Affiliations:** † Department of Drug Chemistry and Technologies Laboratory Affiliated with the Institute Pasteur Italy − Cenci Bolognetti Foundation, Sapienza University of Rome, Rome 00185, Italy; ‡ Department of Biology and Biotechnology “L. Spallanzani”, University of Pavia, Pavia 27100, Italy; § SC Microbiology and Virology, Fondazione IRCCS Ca’ Granda Ospedale Maggiore Policlinico, Milan 20122, Italy; ∥ Pediatric Department, Cystic Fibrosis Pediatric Center, Fondazione IRCCS Ca’ Granda Ospedale Maggiore Policlinico, Milan 20122, Italy; ⊥ Department of Science, University of Roma Tre, Rome 00154, Italy

**Keywords:** virtual screening, FtsZ, antibiotics, antimicrobial resistance, drug discovery

## Abstract

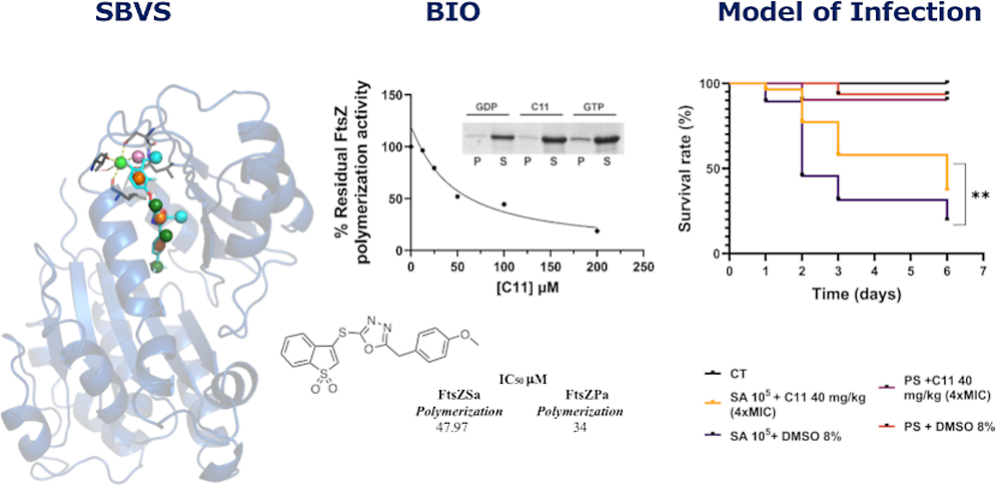

Antimicrobial resistance (AMR) poses a major threat to human health globally. Approximately 5 million deaths were attributed to AMR in 2019, and this figure is predicted to worsen, reaching 10 million deaths by 2050. In the search for new compounds that can tackle AMR, FtsZ inhibitors represent a valuable option. In the present study, a structure-based virtual screening is reported, which led to the identification of derivative **C11** endowed with an excellent minimum inhibitory concentration value of 2 μg/mL against Staphylococcus aureus. Biochemical assays clarified that compound **C11** targets FtsZ by inhibiting its polymerization process. **C11** also showed notable antimicrobial activity against *S. aureus* cystic fibrosis isolates and methicillin-resistant *S. aureus* strains. Derivative **C11** did not show cytotoxicity, while it had a synergistic effect with methicillin. **C11** also showed increased survival in the Galleria mellonella infection model. Lastly, structure–activity relationship and binding mode analyses were reported.

## Introduction

Antimicrobial resistance (AMR) is a serious global health threat. In 2019, it was estimated that around 5 million deaths were attributed to AMR, and this situation is getting worse with an estimation of 10 million deaths annually by 2050.^1^ This phenomenon is mainly due to the extensive misuse of antibiotics which leads to the selection of resistant bacteria.^2^ Although the development of new antibiotics with novel mechanisms of action is crucial to tackle this issue, only 50 new antibacterial drugs are facing clinical evaluation.^3^ Furthermore, only 32 of these are potentially effective against World Health Organization (WHO) priority pathogens.^3^ Among these bacteria, the Enterococcus faecium, *Staphylococcus aureus*, *Klebsiella pneumoniae*, Acinetobacter baumannii, *Pseudomonas aeruginosa,* and *Enterobacter* spp. (ESKAPE) group is particularly worrisome. *S. aureus*, for instance, can persist and adapt to different hostile niches and is equipped with many virulence factors. Methicillin-resistant *S. aureus* (MRSA) strains represent a particular threat to immunocompromised subjects and people with cystic fibrosis.^4^

In the search for new antibiotics with unrelated mechanisms of action, the bacterial cell division machinery represents a valuable target. The division of the parent cell into daughter cells is mainly orchestrated by FtsZ (temperature-sensitive mutant Z). This protein polymerizes, through GTP hydrolysis, to form a single strained filament, linked to the cytoplasmic membrane, leading to a ring-like structure (Z-ring) located in the middle of the parent cell.^5^ The Z-ring serves as a scaffold for the recruitment of other downstream effectors for cell division,^5^ and last, it leads to the final division through the septum constriction along with its invagination and the biosynthesis of peptidoglycan.^5^ The crucial role played by FtsZ makes it a very attractive target. Furthermore, FtsZ is highly conserved in most prokaryotic organisms,^6^ and it is widely demonstrated that its inhibition drives bacteriostatic or bactericidal effects.^7^ Tubulin is the eukaryotic analogue of FtsZ; these proteins share a common structure, nevertheless, the sequence alignment shows that the two proteins have a low sequence identity (less the 20%) and conserved residues localized only in the nucleotide-binding site.^8^

**Figure 1:**
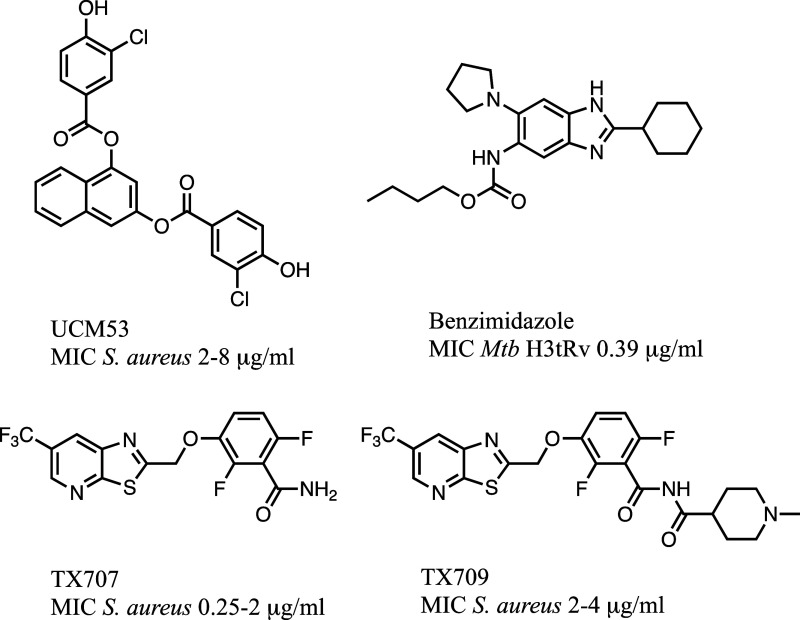
Small molecule FtsZ inhibitors.

All these points confirm the validity of targeting FtsZ: the inhibitors are very effective, and they may have a broad spectrum of activity and are expected to have no cytotoxicity toward eukaryotes. At the state of the art, different FtsZ inhibitors have been reported, including peptides, natural products, and small molecules.^9,10^ Peptides like MciZ (49 residues long)^11^ mainly target the C terminal domain of FtsZ but none of the reported derivatives continued to preclinical or clinical evaluation.^9^ Natural compounds, like Sanguinarine and Berberine, showed promising antimicrobial activity, but they also had significant toxicity toward mammalian cells mainly because of the inhibition of tubulin polymerization.^12−14^ Among the small molecules, three chemical classes showed the most favorable results (Figure 1): (i) the arene-diol digallates (UCM53) targeted the nucleotide-binding site with promising activity toward Gram-positive bacteria but with some cytotoxicity to eukaryotic cells;^15−17^ (ii) the benzimidazole exhibited excellent antibacterial activity against clinical *Mycobacterium tuberculosis* (minimal inhibitory concentration of 0.5–15 μM),^18^ and (iii) benzamide derivatives.^19^ The latter class is exemplified by TX707, which had potent bactericidal activity against several Gram-positive bacteria, including *Bacillus subtilis*, MRSA, and other multidrug resistant (MDR) *S. aureus* in vitro and in vivo. TX709, a prodrug of TX707, is in the clinical phase of evaluation.^19−22^

Despite the effort in the field, the urgency of a new therapeutic armamentarium for MDR strains has made the search for new FtsZ inhibitors a very current topic.

In this scenario, a structure-based virtual screening (VS) campaign was carried out, resulting in the identification of compound **C11** with a promising minimum inhibitory concentration (MIC) of 2 μg/mL against S. aureus. Further biological evaluation confirmed that compound **C11** can impair FtsZ polymerization without being cytotoxic to eukaryotic cells.

The compound was effective against *S. aureus* biofilms, and it also showed a notable antimicrobial activity against cystic fibrosis *S. aureus* clinical isolates and MRSA strains. Moreover, the combination of **C11** with Meropenem disclosed a synergistic effect that restored methicillin susceptibility to MRSA strains. The compound can also increase the survival of Galleria mellonella infected with *S. aureus*. A small group of commercially available **C11** analogues (12 derivatives) was evaluated in vitro and in silico to outline a structure–activity relationship (SAR) and to define the pharmacophoric moieties within the scaffold.

## Results and Discussion

### Screening

To identify new FtsZ inhibitors, a structure-based VS was carried out. FtsZ is characterized by two main binding pockets: (i) the nucleotide binding pocket, bound by GTP/GDP and localized at the polymerization interface, and (ii) an interdomain cleft located at the central core of FtsZ (Figure S1). This pocket has no catalytic activity, and it is not directly involved in polymerization but plays a pivotal role in this process. Thus, this pocket in the *S. aureus* FtsZ protein (FtsZSa; PDB: 4DXD)^23^ was targeted by a docking and pharmacophore-based VS campaign. A database of commercially available compounds (about 5.000.000 molecules) was prefiltered to retain those matching Lipinsky drug-like properties^24^ and to remove unsuitable chemical moieties displaying promiscuous and cytotoxic effects.^25^ The obtained database was docked at the allosteric site of FtsZSa by Glide,^26^ and a pharmacophore model was generated by Phase^27^ merging the information on TX707 SAR.^19−23^ The resulting model included nine features: one H-bond acceptor, two H-bond donors, three hydrophobic, and three aromatic features (Figure S2). This model was used to filter out the docking proposed conformations. In the end, the conformers with the best fitting to the pharmacophore model (a total of 5000) were visually inspected,^28^ and 12 structurally unrelated molecules were purchased for biological evaluation.

### Evaluation of Biological Activities of the Selected Compounds on *S. aureus* FtsZ

To verify whether the selected compounds retained biological activity against bacteria and, in particular, against FtsZSa, the inhibition of bacterial growth, the impairment of the GTPase activity, and, eventually, the inhibition of FtsZ polymerization were assayed (Table S1).

**Figure 2:**
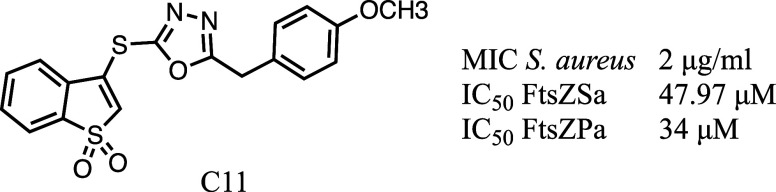
**C11** structure and biological activity.

**Figure 3:**
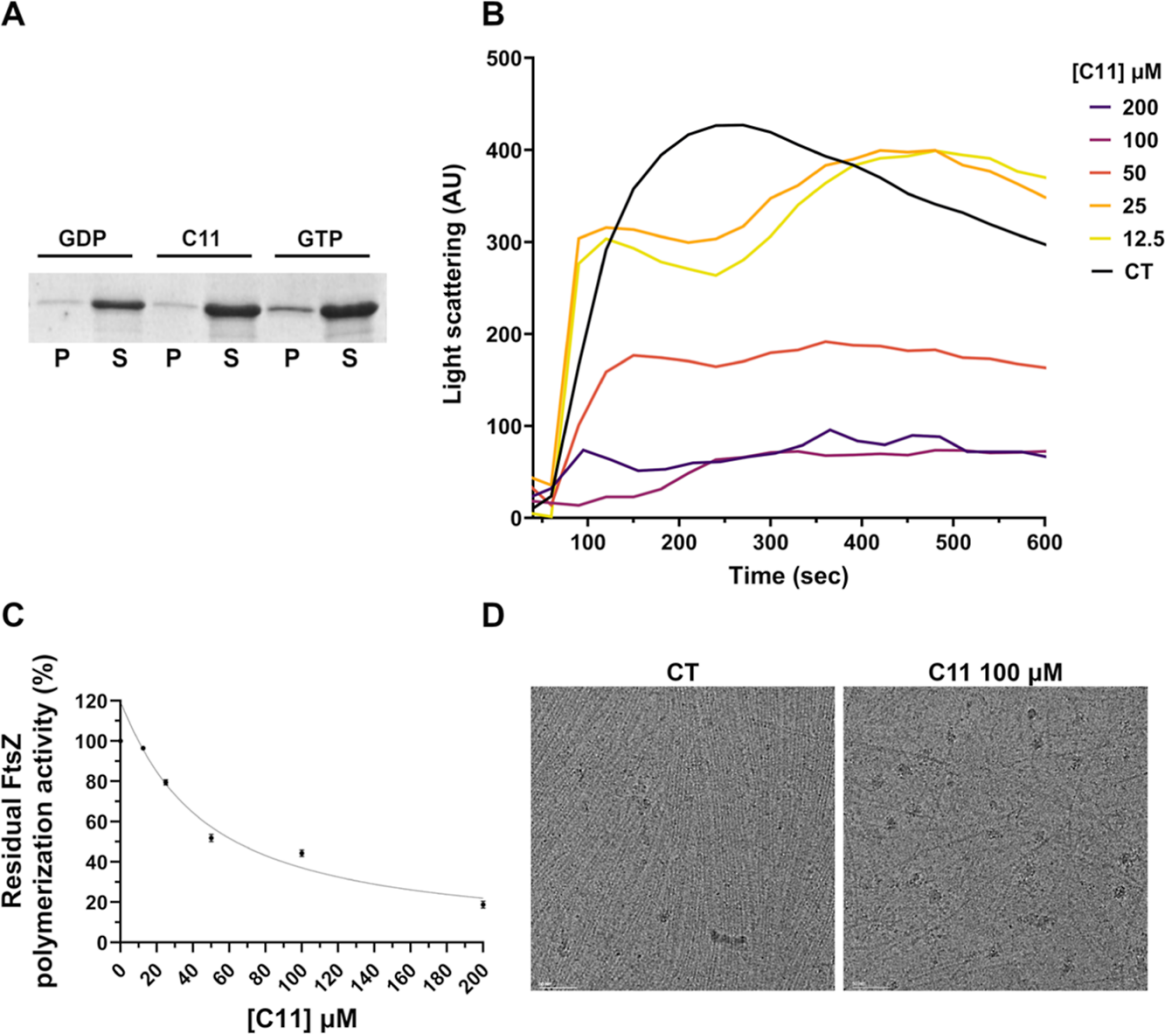
FtsZSa polymerization in the presence of **C11**. (A) Sedimentation assay of FtsZSa in the presence of 100 μM of **C11**. GDP was used as a negative control of the reaction. (P: pellet fraction; S: supernatant fraction). (B) Right-angle light scattering of FtsZSa in the presence of increasing concentration of **C11**. GTP was added after 75 s of incubation. (C) Dose-response curve of FtsZSa residual polymerization activity with **C11**. Data are the mean ± SD of the results from three different replicates. (D) Typical micrograph of FtsZSa at cryo-electron microscope in the absence (left, CT) or in the presence of 100 μM of **C11** (right). The scale bar represents 50 nm. Data and images are representative of the results from three different replicates.

Three compounds (**C3**, **C8**, and **C11**) showed promising inhibitory activity toward FtsZSa GTPase activity. Derivatives **C3** and **C8** showed good inhibition of the GTPase activity with an IC_50_ of 14.6 and 8.4 μM, respectively (Table S1 and Figure S3). Unfortunately, both compounds had MIC values of ≥256 μg/mL when assayed against *S. aureus* ATCC25923 growth (Table S1). On the other hand, **C11** showed a significant inhibition of *S. aureus* ATCC25923 growth with a MIC of 2 μg/mL, but it did not affect the FtsZSa GTPase activity (Table S1). Since FtsZ is characterized by both GTPase and polymerization activity, a sedimentation assay was performed to evaluate the ability of compound **C11** to inhibit FtsZSa polymers formation (Figure 2 and Figure 3A).

Similar to what was previously described for other FtsZ inhibitors, like C109,^29^**C11** had an inhibitory effect on FtsZSa polymerization (Figure 3A). To further evaluate this effect, the kinetics of FtsZSa polymerization was studied using a 90° light scattering measurement. **C11** inhibited the polymerization in a concentration-dependent manner, with an IC_50_ of 47.97 μM (Figure 3B,C). Of note, the presence of **C11** decreased the extent of FtsZSa polymerization and bundling, in a concentration-dependent manner like other previously described inhibitors^30,31^ and decreasing the compound concentration however had an effect on polymer stability (Figure 3B). Moreover, the decrease of polymer formation was also confirmed by cryo-electron microscopy analysis. At a concentration of 100 μM, **C11** significantly impaired the quantity, stability, and bundling of FtsZSa polymers (Figure 3D).

### **C11** Biological Effect and FtsZ Localization

**Figure 4:**
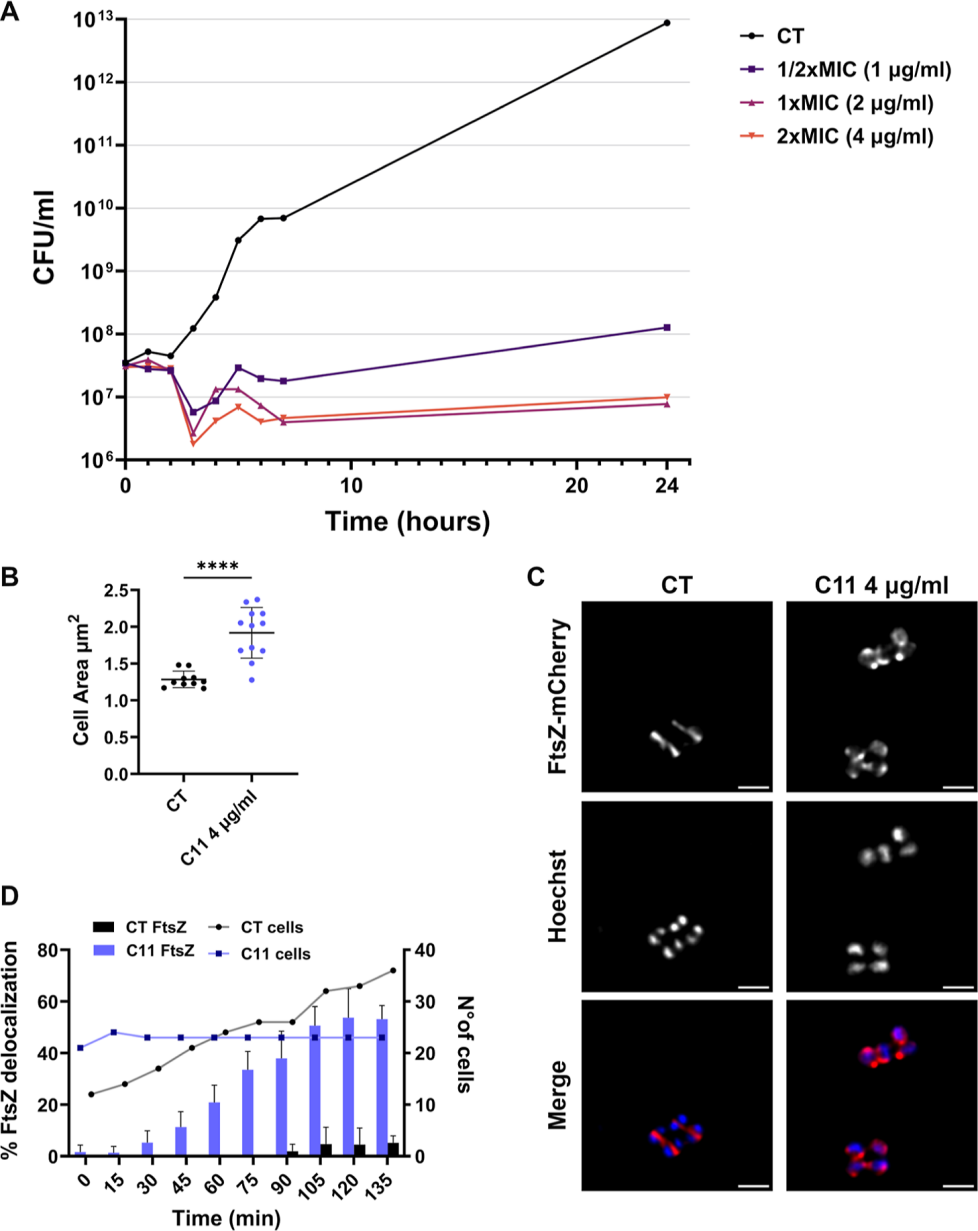
**C11** effect on *S. aureus* ATCC25923 growth. (A) Time-killing curve of *S. aureus* ATCC25923 exposed to **C11**. A *S. aureus* ATCC25923 culture in the exponential phase of growth was split and concentrations of **C11** corresponding to 1/2-, 1-, and 2-fold the MIC value were added. The viable counts were determined at 37 °C over 24 h. (B) Measurements of cell areas of *S. aureus* ATCC25923 untreated and treated with 4 μg/mL of **C11**. Statistically significant differences are indicated (unpaired *t*-test, ****p < 0.0001). (C) Fluorescence microscopy images of *S. aureus* TD276, carrying the FtsZ-mCherry (red) construct, untreated (CT) and treated with 4 μg/mL of **C11**. Cells were stained also with Hoechst (1 μg/mL) (blue). The scale bar corresponds to 2 μm. (D) Graphical representation of the percentage of cells showing FtsZ delocalization (left *y* axis-bars), during growth (right *y* axis-lines) in the absence (black), or in the presence (violet) of 4 μg/mL **C11**, in time-lapse experiments. Data and images are representative of the results of at least three different experiments.

A time–kill curve was constructed using concentrations ranging from 1/2 to 2 times the MIC value (Figure 4A) to assess whether **C11** exerted a bacteriostatic or bactericidal effect on *S. aureus* cells. Bacteriostatic effect was observed using 1- or 2-fold the MIC of **C11**, while a slight regrowth was observed when cells were treated with an amount of compound equal to half of the MIC (Figure 4A). Single-cell time-lapse microscopy of *S. aureus* ATCC25923 demonstrated that the area of the cells is significantly larger in the sample exposed to 2 × MIC of **C11** compound for 5 h compared to untreated cells (Figure 4B). To study FtsZ localization, we used the *S. aureus* TD276 strain expressing the FtsZ-mCherry fusion protein.^32,33^ Against this strain, **C11** retained a MIC of 2 μg/mL. As shown in Figure 4C, in untreated cells, FtsZ was localized in the middle of the cell and formed the Z-ring. In the treated sample, cells were larger and FtsZ was mislocalized, forming different foci which were not functionally competent for cell division (Figure 4C). Moreover, staining of endogenous DNA with Hoechst demonstrated that DNA segregation is not impaired in the treated cells since DNA segregates currently in both treated and untreated cells (Figure 4C). This experiment confirmed once again that FtsZ polymerization is likely the target of the **C11**. Since bacterial growth might be interrupted by the stress conditions imposed by time-lapse microscopy, treated and untreated cells were analyzed for their FtsZ localization in 10 fields. Results showed that the percentage of cells in which FtsZ was delocalized increased over time, and it was significantly higher in **C11**-treated samples as compared to untreated cells (60% vs 4%) (Figure 4D).

### **C11** Activity on Biofilm Formation

**Figure 5:**
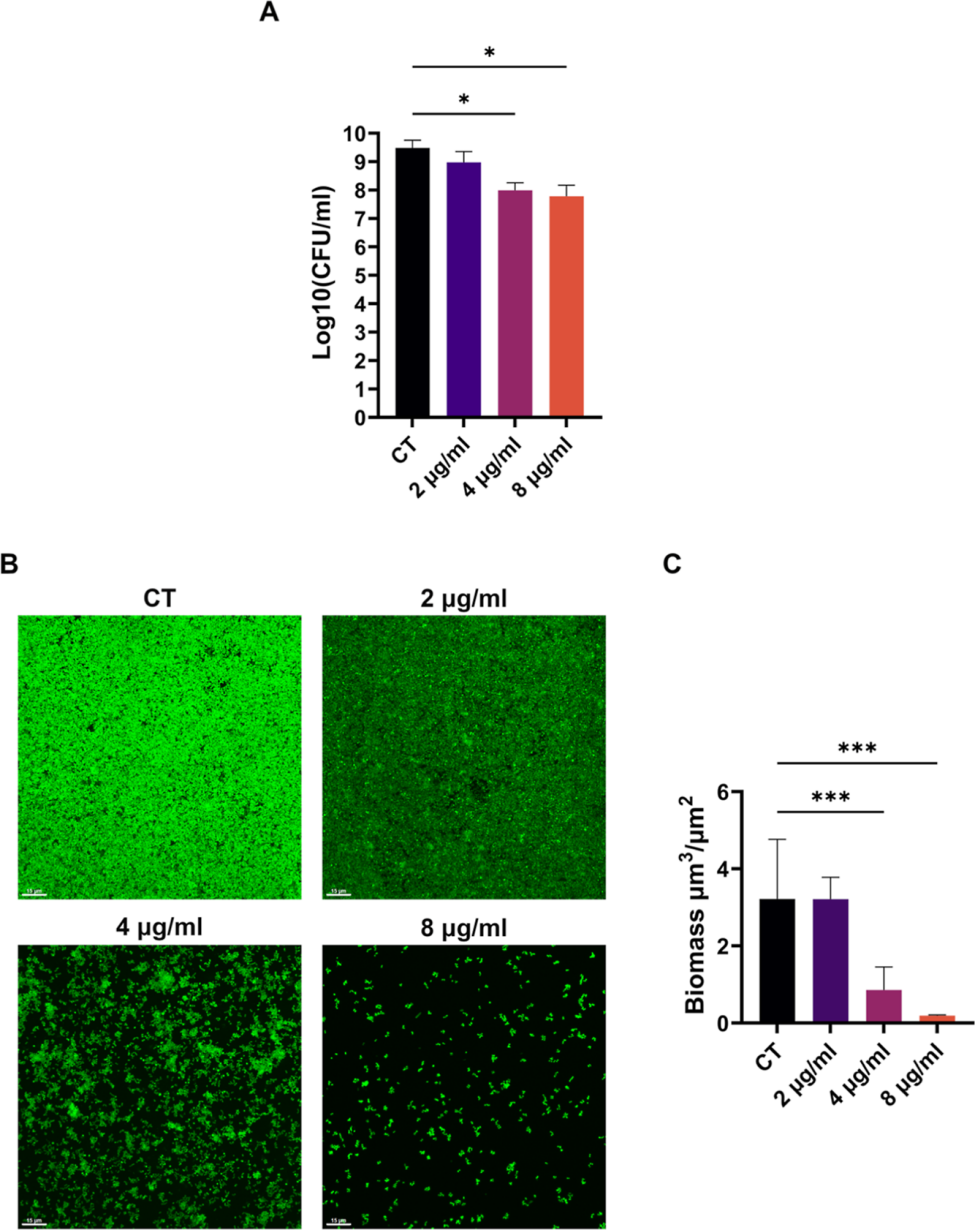
**C11** effect on biofilm inhibition. (A) Bacterial biofilms of *S. aureus* ATCC25923 were grown in the presence of increasing concentrations of **C11**. The results are expressed as CFU/ml recovered after 24 h of incubation. (B) CLSM images of *S. aureus* ATCC25923 biofilms grown in “μ-Slide 4 Well Ibidi treated”. Cells were grown overnight at 37 °C in TSB + 1% glucose with no **C11** (CT), 2, 4, or 8 μg/mL **C11**. Planes at equal distances (0.3 μm) along the *Z*-axis of the biofilm were imaged by CLSM. These 2D images were the maximum projection of the planes. Scale bar represents 15 μm. (C) Analysis of biofilm properties by COMSTAT 2. Measures of the total biomass of the biofilms in the presence of increasing concentrations of **C11**. Data are the mean ± SD of the results from three independent replicates. Images are representative of at least three different experiments. *p < 0.05, ***p < 0.001 (one-way ANOVA test).

*S. aureus* can readily form biofilms enhancing its drug resistance and inducing life-threatening infections in different body compartments.^34^ Hence the ability of **C11** to block biofilm formation and to eradicate formed biofilms was tested using the *S. aureus* ATCC25923 strain. First, the inhibitory activity of the compound was evaluated by CFU counting (Figure 5A), showing that concentrations of 2- or 4-fold the MIC (4–8 μg/mL) were enough to almost completely inhibit biofilm formation (Figure 5A). The structure of the biofilm formed in the presence of increasing concentrations of **C11** was analyzed using confocal laser scanning microscopy (CLSM) and COMSTAT2. These analyses confirmed that **C11** at 2 μg/mL only slightly affected biofilm formation, while it almost completely abolished it at 4 and 8 μg/mL (Figures 5BC and S4). Then the biofilm eradication was also assayed by CFU counting and CLSM. The assays showed that even the lower dose of **C11** (2 × MIC, 4 μg/mL) could reduce the number of viable cells in *S. aureus* ATCC25923 biofilms (Figure S5A); increasing concentrations of **C11** had a strong impact on the biofilm, eradicating the majority of the attached cells (Figure S5B) producing a significant alteration of the biofilm (Figures S5C and S6).

### **C11** Combination with Currently Used Antibiotics

Antibiotic therapies frequently require the combination of multiple antimicrobial drugs to increase efficacy and to overcome antibiotic resistance.^35^ To assess the interaction of **C11** with other compounds, checkerboard assays with currently used antibiotics with different mechanisms of action (Ceftazidime, Erythromycin, Linezolid, Meropenem, Rifampicin, and Tetracycline) were performed. The panel was combined with **C11** and tested against *S. aureus* ATCC25923. While the combination of **C11** with Erythromycin, Linezolid, and Tetracycline had an additive effect, the combination of **C11** with Ceftazidime, Meropenem, and Rifampicin significantly increased their efficacy, showing a synergistic effect (Table S2).

The derivative **C11** was also tested against a panel of clinical isolates obtained from cystic fibrosis patients showing relevant activity against all strains, with MIC values ranging from 2 to 4 μg/mL for the vast majority of them (Tables S3 and S4).

Since some of the tested strains were MRSA, showing resistance to Meropenem (Table S4), the effect of the Meropenem/**C11** combination was assayed against these strains. Results showed that the combination had a strong synergistic effect against the most resistant strains to Meropenem (BG9 and BG10, Table S5 and Figure S7A,B). These data suggest that **C11** has a synergistic effect with β-lactam, as already described for other FtsZ inhibitors, such as PC190723.^22,23^ Moreover, a synergistic effect with Rifampicin was previously described in the case of compounds that induced FtsZ depletion and resulted to be useful to eradicate persistent cells in a chronic biofilm infection.^36^

### **C11** Cytotoxicity Studies

**Figure 6:**
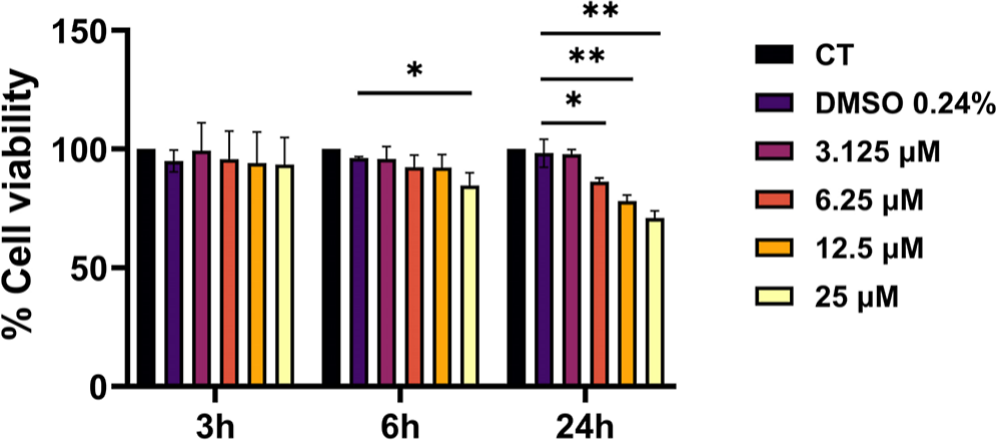
Cytotoxicity of **C11** on A549 using MTT assay. Different **C11** concentrations (3.125–25 μM) and different times of incubation (3, 6, or 24 h) were assayed. Data are presented as mean value ±SD calculated on triplicate experiments. *p < 0.05, **p < 0.01 (unpaired *t*-test).

FtsZ inhibitors might exert a certain toxicity due to their potential inhibition of human tubulin. **C11** cytotoxicity was assayed on mammalian tubulin in vitro and against different human cellular lines.^37^ Compound **C11** decreased tubulin polymerization of 20% at the highest tested concentration (25 μM, 9.6 μg/mL) (Table S6). **C11** cytotoxicity was evaluated by an MTT assay on human A549 pulmonary epithelial cells. In the presence of 25 μM of **C11,** A549 cells showed a viability higher than 90% after 6 h of incubation and the percentage of live cells remained higher than 70% after 24 h (Figure 6). **C11** cytotoxicity was also assayed using CFBEo^–^ cells, mimicking cystic fibrosis lung tissue. In this cell line, with 12.5 μM of compound (4.8 μg/mL) the percentage of live cells was higher than 90% after 6 h of incubation (Figure S8), while in the presence of 25 μM of **C11** the viability decreased to 65% after 6 h of incubation and to 25% after 24 h (Figure S8).

### Efficacy of Compound **C11** in a G. mellonella Infection Model

**Figure 7:**
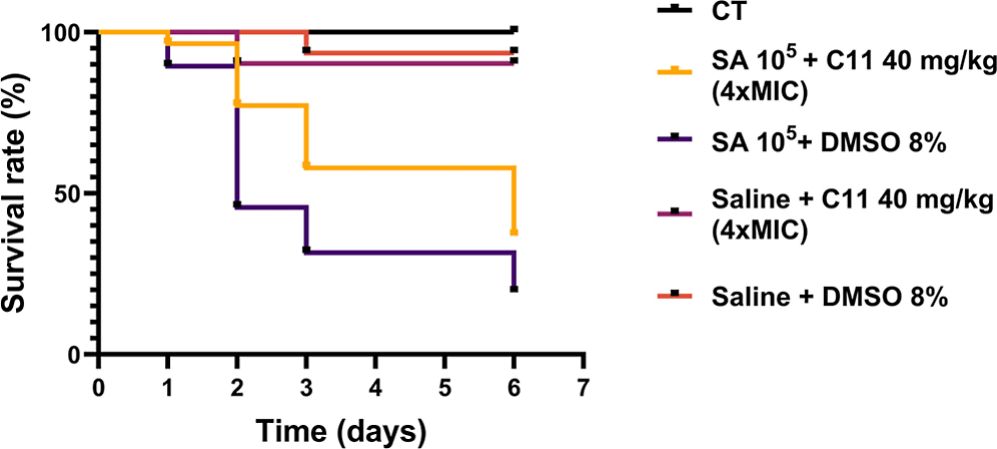
Kaplan–Meier survival curve of G. mellonella larvae infected with *S. aureus* ATCC25923 and treated with 8% DMSO (violet) or **C11** (yellow). As a control, larvae were treated with saline solution and **C11**, saline solution and 8% DMSO (red), or untreated (black). The experiment was performed three times. Statistically significant differences are indicated (Significance level for each time point was evaluated with Fisher’s test: 48 h *p* < 0.001, 72 h *p* < 0.01, and 144 h *p* < 0.05).

A proof-of-concept of the in vivo potential of **C11** in eradicating *S. aureus* infections was given using the well-assessed *G. mellonella* infection model. The larvae were infected with *S. aureus* ATCC25923 and after 2 h treated with 40 mg/kg (4-fold the MIC) of **C11**. Three different groups were used as control: not infected and not treated (CT), injected with saline solution and treated with 40 mg/kg of **C11**, or injected with saline solution and treated with 8% of DMSO. First, results highlighted that **C11** was not toxic for G. mellonella since the moths not infected but treated with **C11** did not show a significant decrease in their survival rate (Figure 7). After infection with *S. aureus* ATCC25923, in the group treated with DMSO, 80% of the larvae died after 6 days. Larvae infected and treated with **C11** (40 mg/kg) had a survival rate of 77% (vs 46%) 2 days postinfection. Three days postinfection, the survival rate was 58% (vs 32%), and after 6 days, the survival rate was still significantly higher than the mock-treated group (Figure 7).

### **C11** Activity against Other ESKAPE Pathogens

The intriguing results prompted us to further investigate the activity of **C11** against a panel of Gram-negative bacteria. The compound was tested against *P. aeruginosa* PAO1, *K. pneumoniae* ATCC13883, and *A. baumannii* ATCC19606. Unfortunately, the measured MIC values were ≥128 μg/mL for all of the tested strains. Consequently, to determine whether **C11** can be a substrate of efflux pumps,^38^ the MIC was evaluated in the presence of different concentrations of the efflux pump inhibitor PaβN.^39^ Indeed, the presence of PAβN conferred to compound **C11** excellent biological activity, with MIC values of 1–16 μg/mL (Table S7).

Since FtsZSa and the FtsZ of *P. aeruginosa* (FtsZPa) share 47% sequence identity, to better understand the results highlighted by the MIC, FtsZPa was expressed, purified, and tested for its activities in the presence of **C11**. Results showed that, as already observed for FtsZSa, **C11** did not interfere with the GTPase activity of FtsZPa, while it can prevent FtsZPa polymerization in a concentration-dependent manner with an IC_50_ of 34 μM (Figure S9A,B).

Overall, these results strongly suggest that, although **C11** may be also active against the FtsZ protein of Gram-negative bacteria, it is extruded out of the cell by efflux pump before it can reach its target.

### Binding Mode Analyses

**Figure 8:**
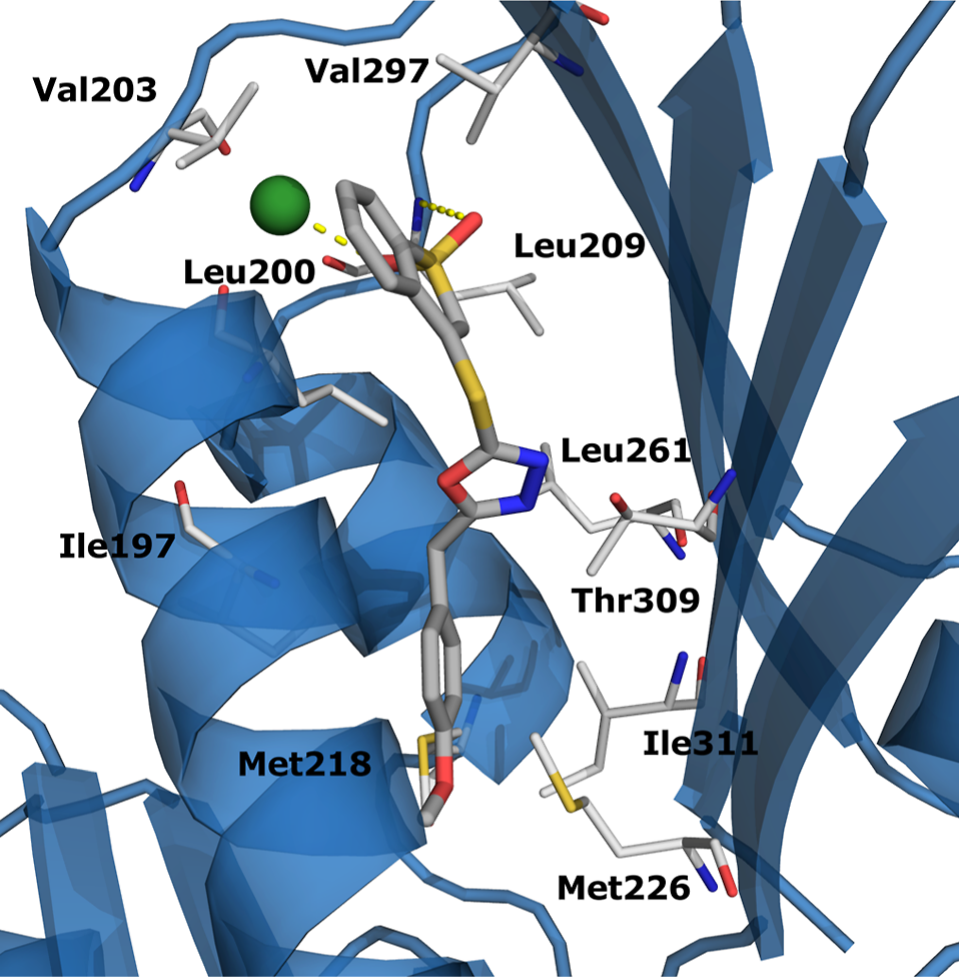
Proposed binding mode of **C11**. The enzyme is reported as blue cartoon, **C11** is depicted as gray sticks. The residues involved in interaction are reported as white lines, polar interactions are reported as yellow dotted lines.

The proposed docking binding mode of **C11** was inspected with the aim of highlighting the key interactions with the target. Overall, the binding with FtsZ was observed to rely largely on hydrophobic contacts: the terminal benzyl ring was involved in interactions with Ile197, Met218, Met226, and Ile311; the oxazole ring had contacts with Leu200, Leu261, and Thr309; and the benzothiophene dioxo ring had hydrophobic interactions with Val203, Leu200, Leu209, and Val297. Polar contacts were also observed involving the sulfone moiety, namely, with the calcium cation and with Leu209 backbone through a H-bond (Figure 8).

### SAR Study

A series of commercially available analogues of **C11** was purchased and tested to draw a SAR. Their ability to inhibit polymerization (Figures S10A,B and S11A–E) and *S. aureus* growth (Table 1) was evaluated. Also, the IC_50_ values against FtsZSa polymerization were determined for the most promising compounds, i.e., **C11.2**, **C11.5**, **C11.7**, and **C11.8**. Compound **C11.1** was selected as a control according to the mild activity on FtsZSa polymerization (Table 1 and Figure S10AB).

**Table 1:**
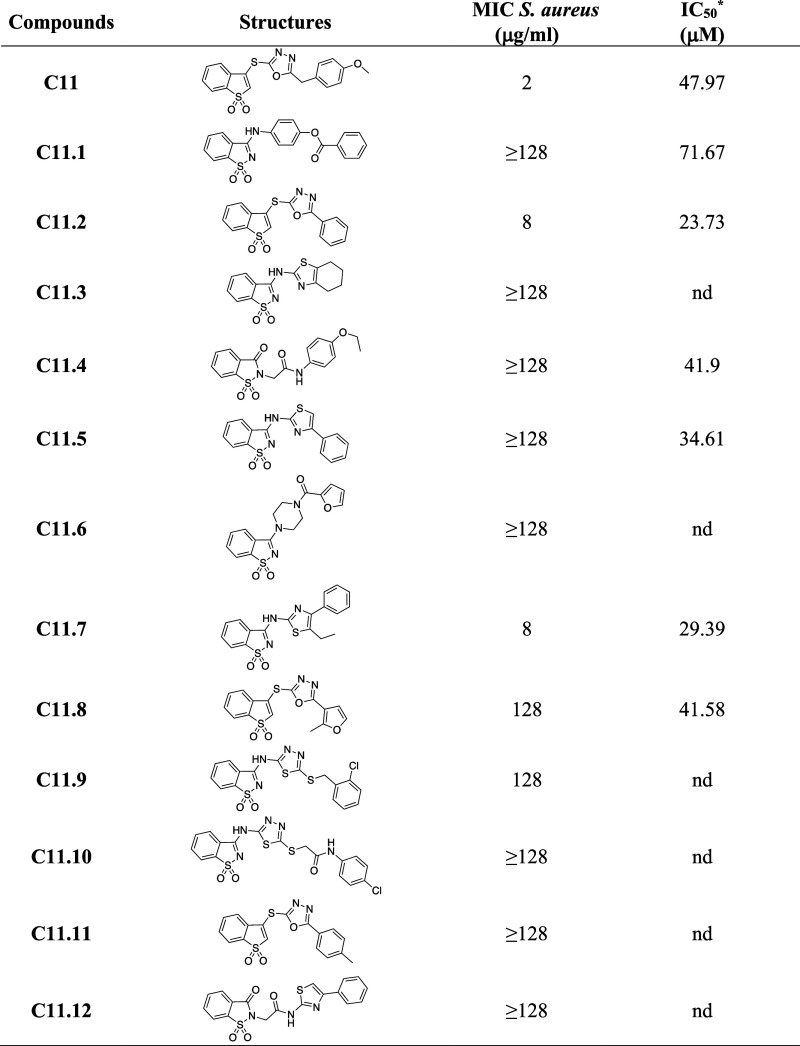
**C11** Derivatives and Their Activities^a^

a Inhibition of FtsZSa polymerization; nd, not determined.

First, it was observed that the methylene bridge between the terminal phenyl ring and the 5-member ring heterocycle could be modified without affecting the binding to the enzyme. Indeed, planar and shortened derivatives without this bridge atom (**C11.2, C11.5**, **C11.7**, and **C11.8**) showed lower IC_50_ values than the lead compound **C11**, and derivative **C11.2** had the lowest IC_50_ value of 23.73 μM (Table 1, Figures S10A,B and S11A–E). Another interesting observation was that the sulfur atom bridging the two aromatic rings could be changed to nitrogen with marginal effect on the inhibition of the polymerization (compare **C11** and **C11.5**). Also the introduction of a nitrogen atom in the thiophane dioxo ring did not affect the IC_50_. However, none of the assayed derivatives had MIC values lower than those of the lead compound **C11**, despite the improved inhibition of FtsZ polymerization. Only derivatives **C11.2** and **C11.7** retained the ability to inhibit *S. aureus* growth in the same range of concentrations of the lead compound.

Lastly, the introduction of a short alkyl ramification at the thiazole ring (compare derivatives **C11.5** and **C11.7**) improved the biological activity. Indeed, compound **C11.7** had a slightly better inhibition of the FtsZ polymerization than **C11.5** (IC_50_ 29.39 μM and IC_50_ 34.61 μM, respectively) but a marked improvement of the antibacterial activity (MIC 8 and ≥ 128 μg/mL). This result might be related to an increased membrane permeability of the compound.^40^

## Conclusions

The lack of new solutions against the spread of antimicrobial resistance prompts the research toward different strategies to identify new active molecules, with the exploration of new and essential pathways being a valuable option for this aim. In this work, a structure-based VS on the essential cell division protein FtsZ led to the identification of derivative **C11**, with a notable antimicrobial activity against *S. aureus*, cystic fibrosis *S. aureus* clinical isolates, and MRSA strains. **C11** mechanism of action is based on impairing FtsZ polymerization. Furthermore, this molecule has very low toxicity. The efficacy of **C11** was further studied in the G. mellonella infection model, confirming that it can increase the survival of larvae infected with *S. aureus*. A series of **C11** analogues were assayed to outline a SAR and together with a docking experiment, essential interactions with the enzyme were highlighted. Taken together, the reported results indicate that **C11** is a promising drug candidate with great potential to be further structurally optimized in order to improve its efficacy and to broaden the spectrum of action.

## Methods

All details of the molecular modeling, chemistry, and biological assays are reported in the Supporting Information.

## Supporting Information Available

FtsZ domains and binding sites; proposed pharmacophore model; list and inhibitory activity of the compounds selected from the virtual screening; dose-response curves of residual FtsZSa GTPase activity in the presence of C3, C8, or C11; analysis of biofilm inhibition by COMSTAT2; C11 effect on biofilm eradication; analysis of biofilm eradication by COMSTAT2; MIC values of different antibiotics and C11, their combination, and FICI values; bacterial strains used in this study; MIC of C11 for cystic fibrosis clinical isolates; MIC values of Meropenem and C11, their combination and the FICI values, against different MRSA clinical isolates; tubulin polymerization assay; checkerboard assay of BG9 and BG10 strains; cytotoxicity of C11 to CFBE41o- using MTT assay; MIC of P. aeruginosa, K. pneumoniae, and A. baumannii against C11 in combination with PaBN; FtsZPa polymerization assay in the presence of the C11 derivatives; FtsZSa sedimentation assay densiometric analyses of the C11 derivatives; dose-response curves of FtsZSa residual polymerization activity in the presence of C11 analogues; and ^1^H NMR spectrum and ^13^C NMR spectrum of compound C11 (PDF)
