# Supplementary material for: Identification of a New FtsZ Inhibitor by Virtual Screening, Mechanistic Insights, and Structure–Activity Relationship Analyses
Source: ACS Infect Dis. 2025 Mar 18;11(4):998–1007. doi: 10.1021/acsinfecdis.4c01045 (PMC11998009; doi:10.1021/acsinfecdis.4c01045)
Supplement: Supplementary file 1 — id4c01045_si_001.pdf [file id4c01045_si_001.pdf]

Supporting Information

Identification of new FtsZ inhibitor by virtual screening, mechanistic insight and SAR analyses

Pietro Sciò[1+], Viola Camilla Scoffone[2+], Anastasia Parisi[1], Marianna Bufano[1], Martina Caneva[2], Gabriele Trespidi[2], Samuele Irudal[2], Giulia Barbieri[2], Lisa Cariani[3], Beatrice Silvia Orena[3], Valeria Daccò[4], Francesco Imperi[5], Silvia Buroni[2] and Antonio Coluccia[1]*

[1]Department of Drug Chemistry and Technologies Laboratory affiliated with the Institute Pasteur Italy – Cenci Bolognetti Foundation, Sapienza University of Rome, 00185 Rome, Italy.

[2]Department of Biology and Biotechnology "L. Spallanzani", University of Pavia, 27100 Pavia, Italy.

[3]SC Microbiology and Virology, Fondazione IRCCS Ca' Granda Ospedale Maggiore Policlinico, 20122 Milan, Italy.

[4]Pediatric Department, Cystic Fibrosis Pediatric Center, Fondazione IRCCS Ca' Granda Ospedale Maggiore Policlinico, 20122 Milan, Italy.

[5]Department of Science, University of Roma Tre, 00154 Rome, Italy.

[+] These authors contributed equally to the work

*Corresponding author: Antonio Coluccia, antonio.coluccia@uniroma1.it, Piazzale A. Moro, 5, 00185 Rome, Italy.

Table of contents

Figure S1 FtsZ domains and binding sites
Figure S2. Proposed Pharmacophore model
Table S1. List and inhibitory activity of the compounds selected from the virtual screening.
Figure S3. Dose-response curves of residual FtsZSa GTPase activity in the presence of **C3**, **C8** or **C11**.
Figure S4. Analysis of biofilm inhibition by COMSTAT2
Figure S5. **C11** effect on biofilm eradication.
Figure S6. Analysis of biofilm eradication by COMSTAT2
Table S2. MIC values of different antibiotics and **C11**, their combination, and FICI values
Table S3. Bacterial strains used in this study
Table S4. MIC of **C11** for CF clinical isolates.
Table S5. MIC values of Meropenem and **C11**, their combination and the FICI values, against different MRSA clinical isolates.
Table S6. Tubulin polymerization assay
Figure S7. Checkerboard assay of BG9 and BG10 strains.
Figure S8. Cytotoxicity of **C11** to CFBE41o- using MTT assay
Table S7. MIC of *P. aeruginosa*, *K. pneumoniae* and *A. baumannii* against **C11** in combination with PaβN
Figure S9. FtsZPa polymerization assay in the presence of the **C11** derivatives
Figure S10. FtsZSa sedimentation assay densiometric analyses of the **C11** derivatives
Figure S11. Dose-response curves of FtsZSa residual polymerization activity in the presence of **C11** analogues.
Figure S12. ^{1}H NMR spectrum of compound **C11**
Figure S13. ^{13}C NMR spectrum of compound **C11**
Methods
References

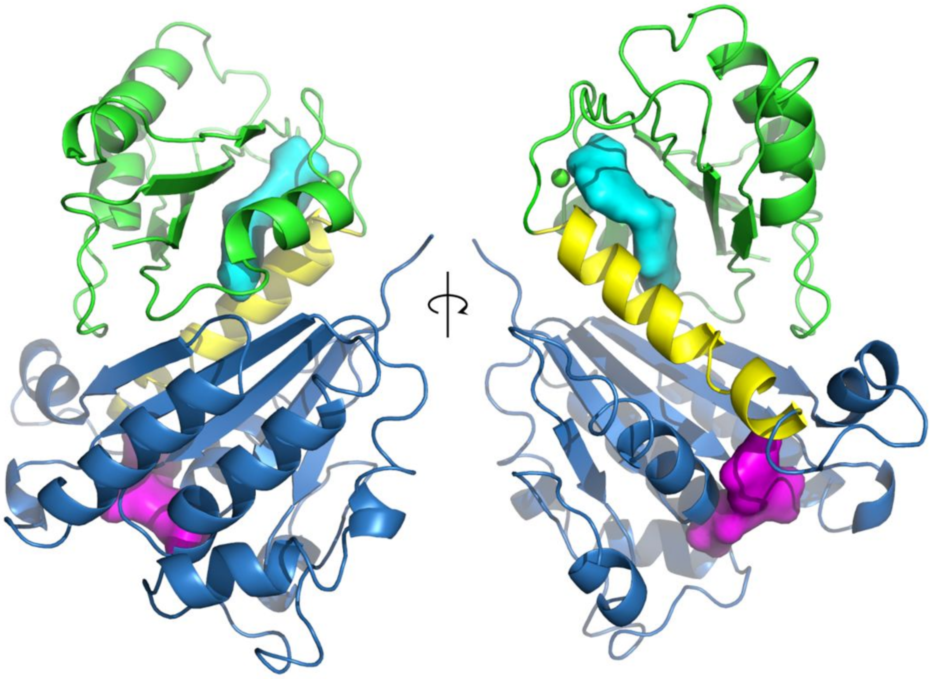

Figure S1. Structure of FtsZ (PDB 4DXD). N-terminal domain is reported in blue, H7 is depicted in yellow and C-terminal domain is reported in green. The ATP binding site is showed as magenta surface and the allosteric site is depicted as cyan surface.

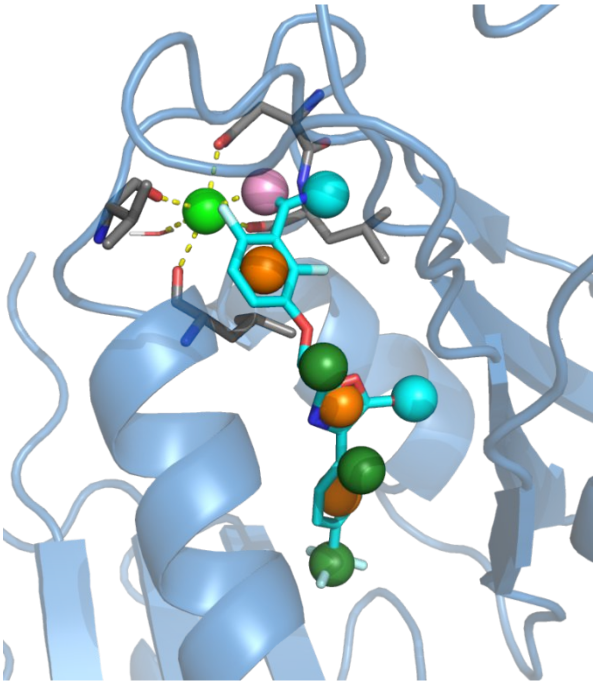

Figure S2. Proposed Pharmacophore model. Queries are reported with spheres: pink for H-bond acceptor, cyan for H-bond donor, forest green for hydrophobic and orange for aromatic features. Protein is reported as blues cartoon, reference compound TX707 is reported as cyan stick, calcium cation is depicted as green sphere, residues involved in cation binding are reported as grey stick. Polar contacts are depicted as yellow lines.

Table S1. List and inhibitory activity of the compounds selected from the virtual screening.

Compound	Vendors code[a]	MIC *S. aureus* (µg/ml)[b]	Residual GTPase activity[c]	IC_{50} (GTPase activity)
Negative Control		-	100 %	-
C109[1]		2–4	23.7 %	1.5 µM
C1	F2775-0365	≥ 256	78.5 %	-
C2	F6521-1780	≥ 256	35.5 %	-
C3	F6523-2830	≥ 256	16.2 %	14.6 µM
C4	F6523-2830	≥ 256	71 %	-
C5	F5791-2180	≥ 256	95.3%	-
C6	F6709-5253	≥ 256	92.8 %	-
C7	AN-153/I141279	≥ 256	107.2 %	-
C8	AN-329/43449396	≥ 256	11.8 %	8.4 µM
C9	AG-690/15433975	≥ 256	34.3 %	-
C10	AT-084/J173614	≥ 256	86.6 %	-
C11	AP-853/J241766	2	82.2 %	-
C12	AG-205/1207517	≥ 256	17.4 %	60 µM

[a]Vendors code for tested compounds. C1 - C6 were purchased from Lifechemicals[2], C7 – C12 were purchased from Specs[3]. [b]MIC for *S. aureus* ATCC25923. [c]Determined using the *S. aureus* FtsZ protein.

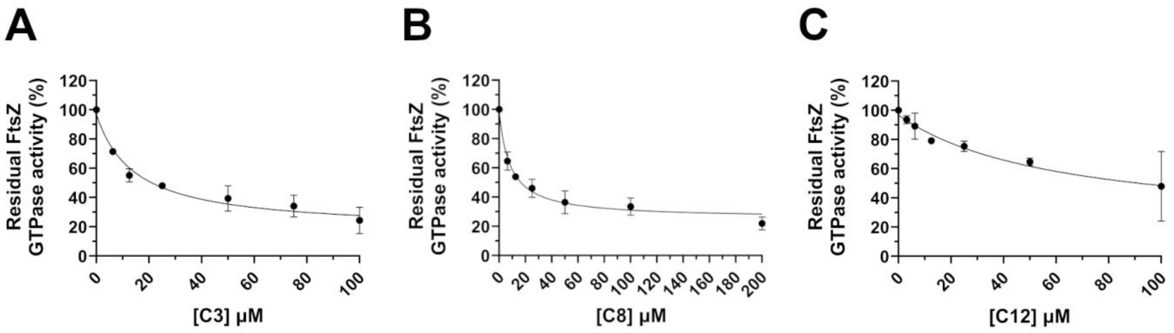

Figure S3. Dose-response curves of residual FtsZSa GTPase activity in the presence of (A) **C3**, (B) **C8** or (C) **C11**.

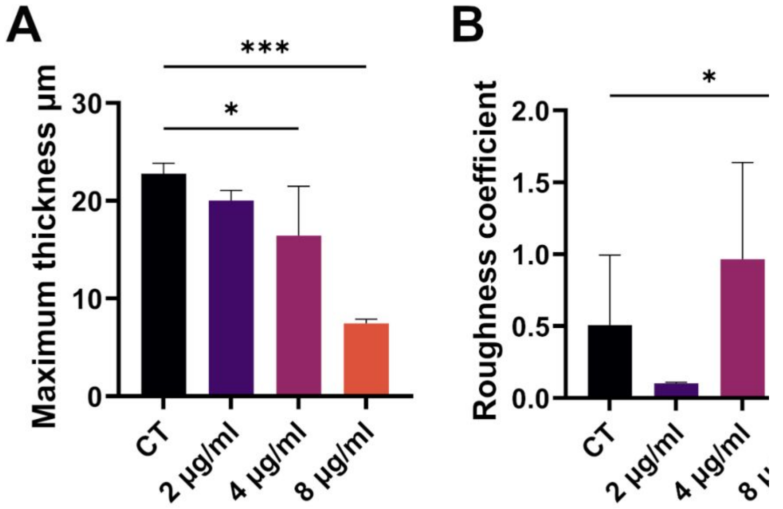

Figure S4. Analysis of biofilm inhibition by COMSTAT2. (**A**) Measures of the maximum thickness of biofilms treated with increasing concentration of **C11**, obtained with COMSTAT2 from CLSM images. (**B**) Measures of the roughness of the biofilms treated with increasing concentration of **C11**, obtained with COMSTAT2 from CLSM images. CT is the untreated biofilm. Data are the mean ± SD of the results from three independent replicates. *p < 0.05; *** p < 0.001 (one-way ANOVA test).

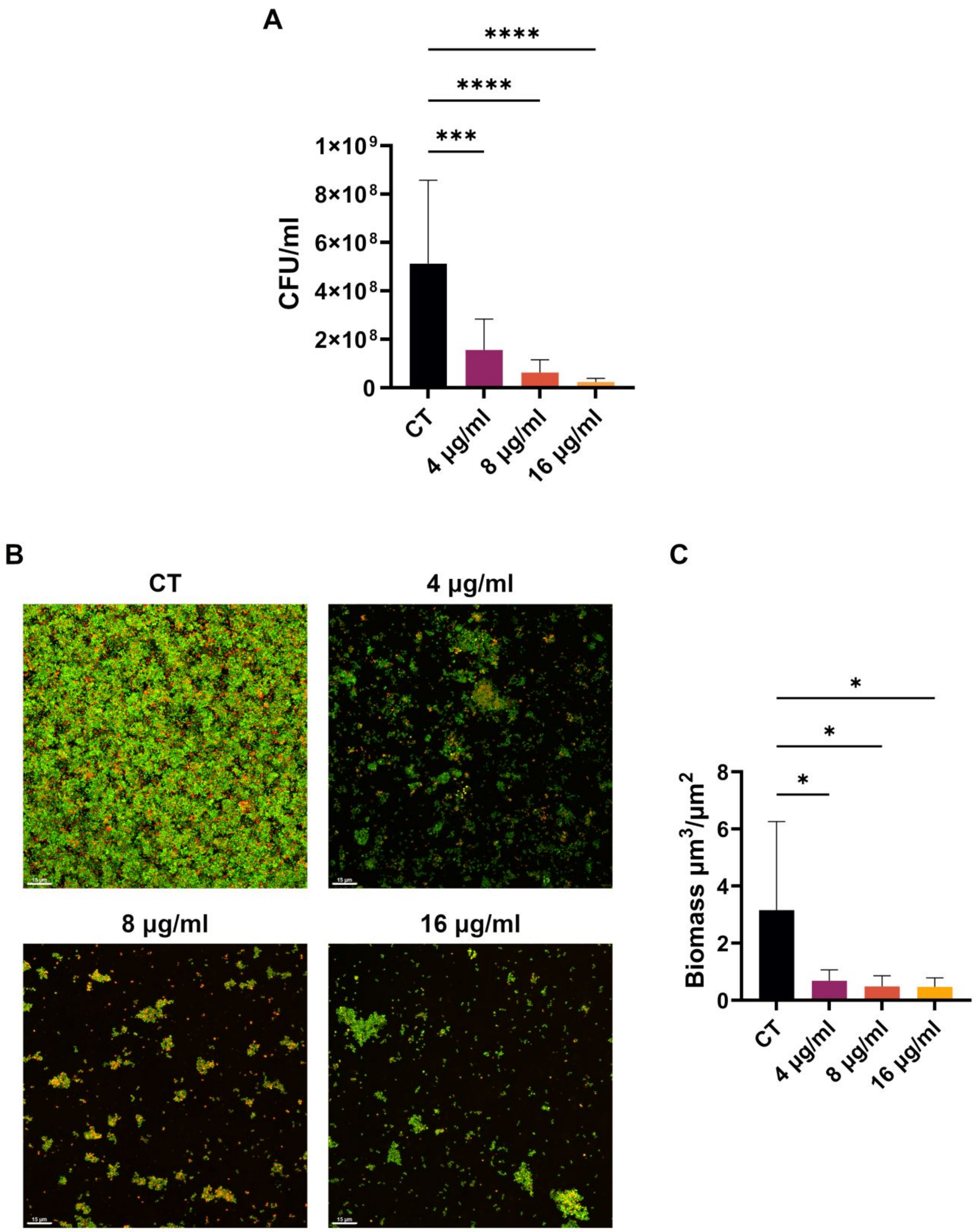

Figure S5. C11 effect on biofilm eradication. (**A**) Bacterial biofilms of *S. aureus* ATCC25923 were grown for 24 hours and then treated for 24 hours with increasing concentrations of **C11**. The results are expressed as CFU/ml recovered at the end of the treatment. (**B**) CLSM images of *S. aureus* ATCC25923 biofilms grown in "µ-Slide 4 Well Ibidi treated". Cells were grown overnight at 37°C in TSB + 1% glucose and after 24 hours were treated with 4 µg/ml, 8 µg/ml or 16 µg/ml of **C11**; CT is the untreated sample. Planes at equal distances (0.3 µm) along the Z-axis of the biofilm were imaged by CLSM. These 2D images were the maximum projection of the planes. Scale bar represents 15 µm. (**C**) Analysis of biofilm properties by COMSTAT 2. Measures of the total biomass of the biofilms in the presence of increasing concentrations of **C11**. Data are the mean ± SD of the results from three independent replicates. Images are representative of at least three different experiments. $^*p < 0.05$, $^{***}p < 0.001$, $^{****}p<0.0001$ (one-way ANOVA test).

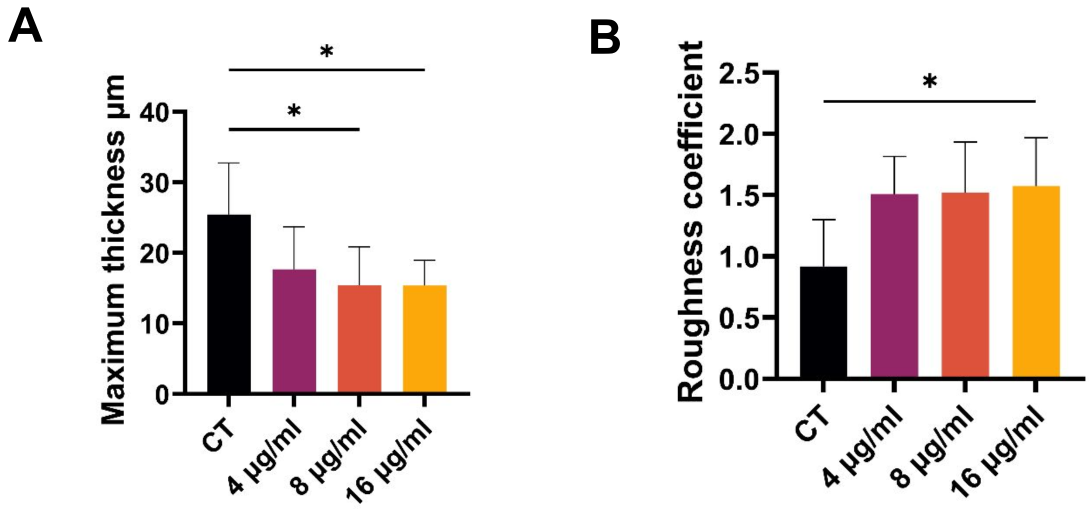

Figure S6. Analysis of biofilm eradication by COMSTAT2.
(**A**) Measures of the maximum thickness of biofilms treated with increasing concentration of **C11**, obtained with COMSTAT2 from CLSM images. (**B**) Measures of the roughness of the biofilms treated with increasing concentration of C11, obtained with COMSTAT2 from CLSM images. CT is the untreated biofilm. Data are the mean ± SD of the results from three independent replicates. *p < 0.05(one-way ANOVA test).

Table S2. MIC values of different antibiotics and **C11**, their combination, and FICI values.

	***S. aureus* ATCC25923**		
**Antibiotics**	**MIC (µg/ml) Single drugs**	**MIC (µg/ml) combination**	**FICI[a]**
Ceftazidime/C11	16/2	4/0.5	Synergy (0.50)
Erythromycin/C11	0.5/2	0.06/1	Additive (0.62)
Linezolid/C11	2/2	0.5/1	Additive (0.75)
Meropenem/C11	0.25/2	0.03/0.5	Synergy (0.37)
Rifampicin/C11	0.03/2	0.007/0.25	Synergy (0.36)
Tetracycline/C11	0.5/2	0.25/1	Additive (1)

[a]: FICI Fractional Inhibitory Concentration Index. The results indicate synergism when the corresponding FICI ≤ 0.5; additivity when 0.5 < FICI ≤ 1, indifference when 1 < FICI ≤ 4 and antagonism when the FICI > 4.

Table S3. Strains used in this study

Strain	Source
S. aureus ATCC 25923	Laboratory collection
S. aureus TD276	Do et al., 2020[4]
S. aureus BG1	Bambino Gesù hospital Rome[5]
S. aureus BG2	Bambino Gesù hospital Rome[5]
S. aureus BG3	Bambino Gesù hospital Rome[5]
S. aureus BG4	Bambino Gesù hospital Rome[5]
S. aureus BG5	Bambino Gesù hospital Rome[5]
S. aureus BG6 MRSA	Bambino Gesù hospital Rome[5]
S. aureus BG7 MRSA SMV	Bambino Gesù hospital Rome[5]
S. aureus BG8 MRSA	Bambino Gesù hospital Rome[5]
S. aureus BG9 MRSA	Bambino Gesù hospital Rome[5]
S. aureus BG10 MRSA	Bambino Gesù hospital Rome[5]
S. aureus SA1	Ospedale Maggiore Policlinico Milan
S. aureus SA2	Ospedale Maggiore Policlinico Milan
S. aureus SA3	Ospedale Maggiore Policlinico Milan
S. aureus SA4	Ospedale Maggiore Policlinico Milan
S. aureus SA5	Ospedale Maggiore Policlinico Milan
S. aureus SA6	Ospedale Maggiore Policlinico Milan
S. aureus SA7	Ospedale Maggiore Policlinico Milan
S. aureus SA8	Ospedale Maggiore Policlinico Milan
S. aureus SA9	Ospedale Maggiore Policlinico Milan
S. aureus SA10	Ospedale Maggiore Policlinico Milan
S. aureus SA11	Ospedale Maggiore Policlinico Milan
S. aureus SA12	Ospedale Maggiore Policlinico Milan
S. aureus SA13	Ospedale Maggiore Policlinico Milan
S. aureus SA14	Ospedale Maggiore Policlinico Milan
S. aureus SA15	Ospedale Maggiore Policlinico Milan
S. aureus SA16	Ospedale Maggiore Policlinico Milan
S. aureus SA17	Ospedale Maggiore Policlinico Milan
S. aureus SA18	Ospedale Maggiore Policlinico Milan
S. aureus SA19	Ospedale Maggiore Policlinico Milan
S. aureus SA20	Ospedale Maggiore Policlinico Milan
S. aureus SA21	Ospedale Maggiore Policlinico Milan
S. aureus SA22	Ospedale Maggiore Policlinico Milan
S. aureus SA23	Ospedale Maggiore Policlinico Milan
S. aureus SA24	Ospedale Maggiore Policlinico Milan
S. aureus SA25	Ospedale Maggiore Policlinico Milan
P. aeruginosa PAO1	Laboratory collection
K. pneuomoniae ATCC13883	Laboratory collection
A. baumannii ATCC19606	Laboratory collection
E. coli BL21DE3	Laboratory collection

Bacterial strains were obtained by Cystic Fibrosis patients upon informed (oral) consent. Samples were collected at Fondazione IRCCS Ca' Granda Ospedale Maggiore Policlinico hospitals and assayed at University of Pavia as stated by an agreement between the hospitals and the for the university for the duration the study. Bacterial strains were collected using Microbank®, a cryovial system incorporating treated beads and a special cryopreservative solution.

Table S4. MIC of **C11** for CF clinical isolates.

**Strain**	MIC (µg/ml) **C11**	**Ciprofloxacin***	**Meropenem***
S. aureus ATCC 25923	2	0.5	0.25
S. aureus BG1	2	1	0.5
S. aureus BG2	4	4	0.5
S. aureus BG3	4	0.5	0.25
S. aureus BG4	8	2	0.25
S. aureus BG5	8	1	0.25
S. aureus BG6 MRSA	2	8	4
S. aureus BG7 MRSA smv	4	2	2
S. aureus BG8 MRSA	2	0.25	2
S. aureus BG9 MRSA	2	256	128
S. aureus BG10 MRSA	2	64	16
S. aureus SA1	4	0.5	
S. aureus SA2	4	0.5	
S. aureus SA3	2	0.5	
S. aureus SA4	2	0.5	
S. aureus SA5	4	1	
S. aureus SA6	2	0.5	
S. aureus SA7	2	0.5	
S. aureus SA8	2	0.25	
S. aureus SA9	4	0.5	
S. aureus SA10	2	1	
S. aureus SA11	4	8	
S. aureus SA12	4	0.25	
S. aureus SA13	4	0.5	
S. aureus SA14	2	0.5	
S. aureus SA15	4	1	
S. aureus SA16	4	0.5	
S. aureus SA17	2	1	
S. aureus SA18	4	64	
S. aureus SA19	2	32	
S. aureus SA20	4	0.5	
S. aureus SA21	2	128	
S. aureus SA22	2	2	
S. aureus SA23	4	0.5	
S. aureus SA24	2	128	
S. aureus SA25	2	0.5	

*:Ciprofloxacin and Meropenem were used as controls.

Table S5. MIC values of Meropenem and **C11**, their combination and the FICI values, against different MRSA clinical isolates.

	MIC (µg/mL)		
**Strains**	**Single drugs (Meropenem/C11)**	**Combination (Meropenem/C11)**	**FICI[a]**
BG8	2/2	0.128/1	Additive (0.56)
BG9	128/2	0.5/0.5	Synergy (0.25)
BG10	16/2	2/0.5	Synergy (0.37)

[a]: The results indicate synergism when the corresponding FICI ≤ 0.5; additivity 0.5 < FICI ≤ 1, indifference 1 < FICI ≤ 4 and antagonism the FICI > 4.

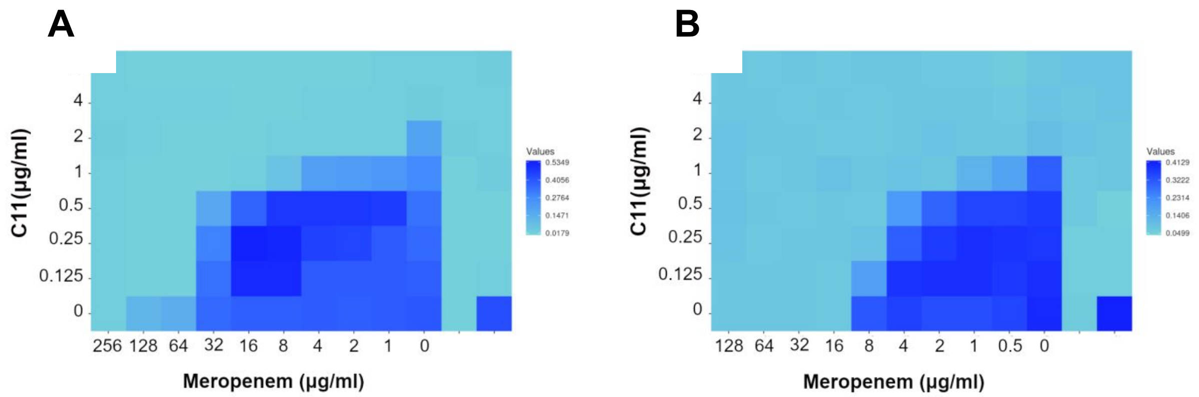

Figure S7. Checkerboard assay of BG9 (A) and BG10 (B) strains. **C11** was combined with Meropenem. Optical density at 600 nm was compared to the growth control (the blue square on the bottom right).

Table S6. Tubulin polymerization assay

Compound	Vmax (mOD/min)
Positive control*	3.78
Paclitaxel (10 µM)**	21.45
C11 (5 µM)	3.5
C11 (10 µM)	3
C11 (25µM)	2.8

Compounds that interact with tubulin alter the polymerization phases, increasing or decreasing the Vmax of the polymerization kinetic.
*DMSO was used as positive control
** The antimitotic drug paclitaxel was used as a control at a final concentration of 10 µM. This compound eliminates the nucleation phase and enhances the Vmax.

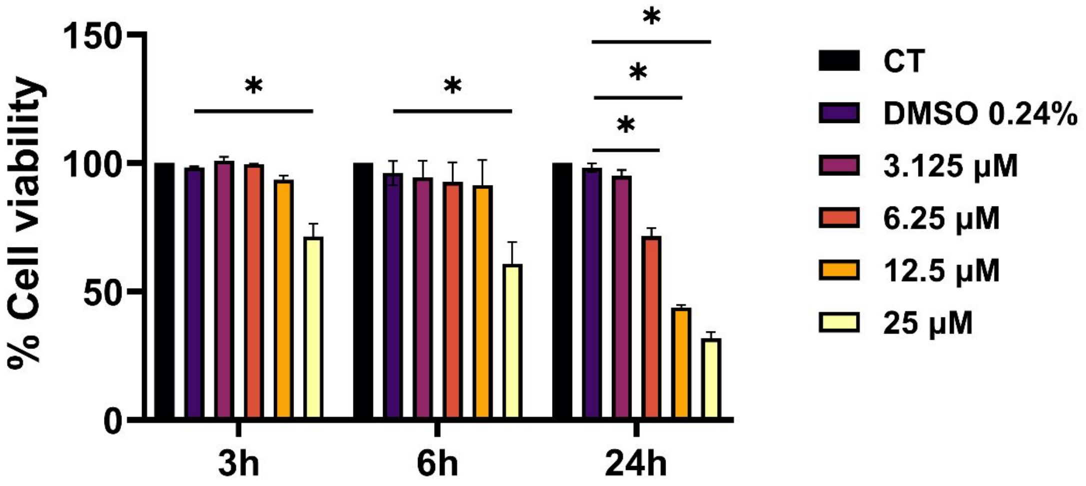

Figure S8. Cytotoxicity of **C11** to CFBE41o- using MTT assay.
Different **C11** concentrations (3.125-25 µM) and different times of incubation (3, 6 or 24 hours) were assayed. Data are presented as mean value ± SD calculated on triplicate experiments. *$p < 0.05$ (unpaired t-test).

Table S7. MIC of *P. aeruginosa*, *K. pneumoniae* and *A. baumannii* against **C11** in combination with PaβN.

	**MIC (µg/ml)**		
**Compound**	***P. aeruginosa*** **PAO1**	***K. pneumoniae*** **ATCC13883**	***A. baumannii*** **ATCC19606**
C11	≥128	≥128	≥128
C11 + PaβN (128 µg/ml)	1	8	nd
C11 + PaβN (64 µg/ml)	16	nd	1

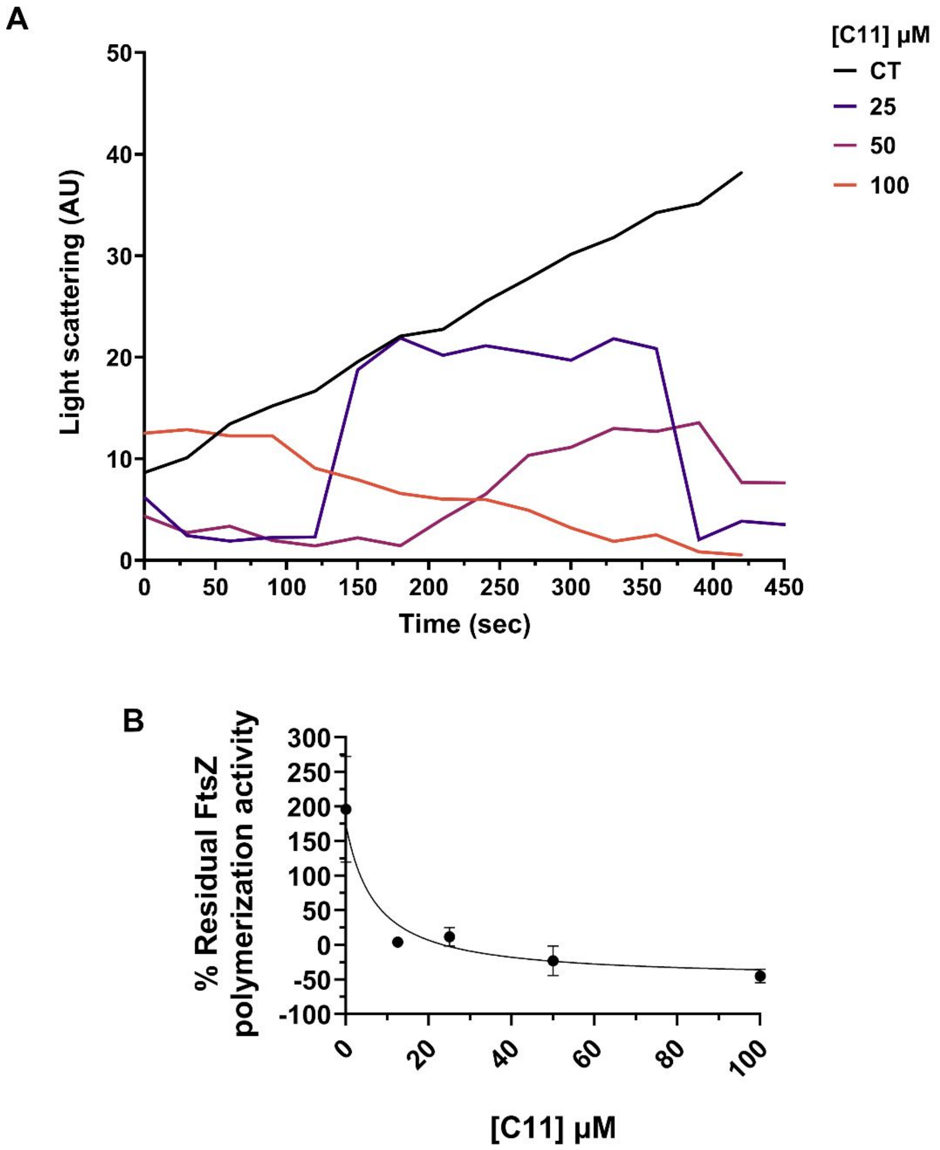

Figure S9. FtsZPa polymerization in the presence of **C11**. (**A**) Right-angle light scattering of FtsZPa in the presence of increasing concentration of **C11**. GTP was added after 25 seconds of incubation. (B) Dose-response curve of FtsZPa residual polymerization activity in the presence of **C11**.

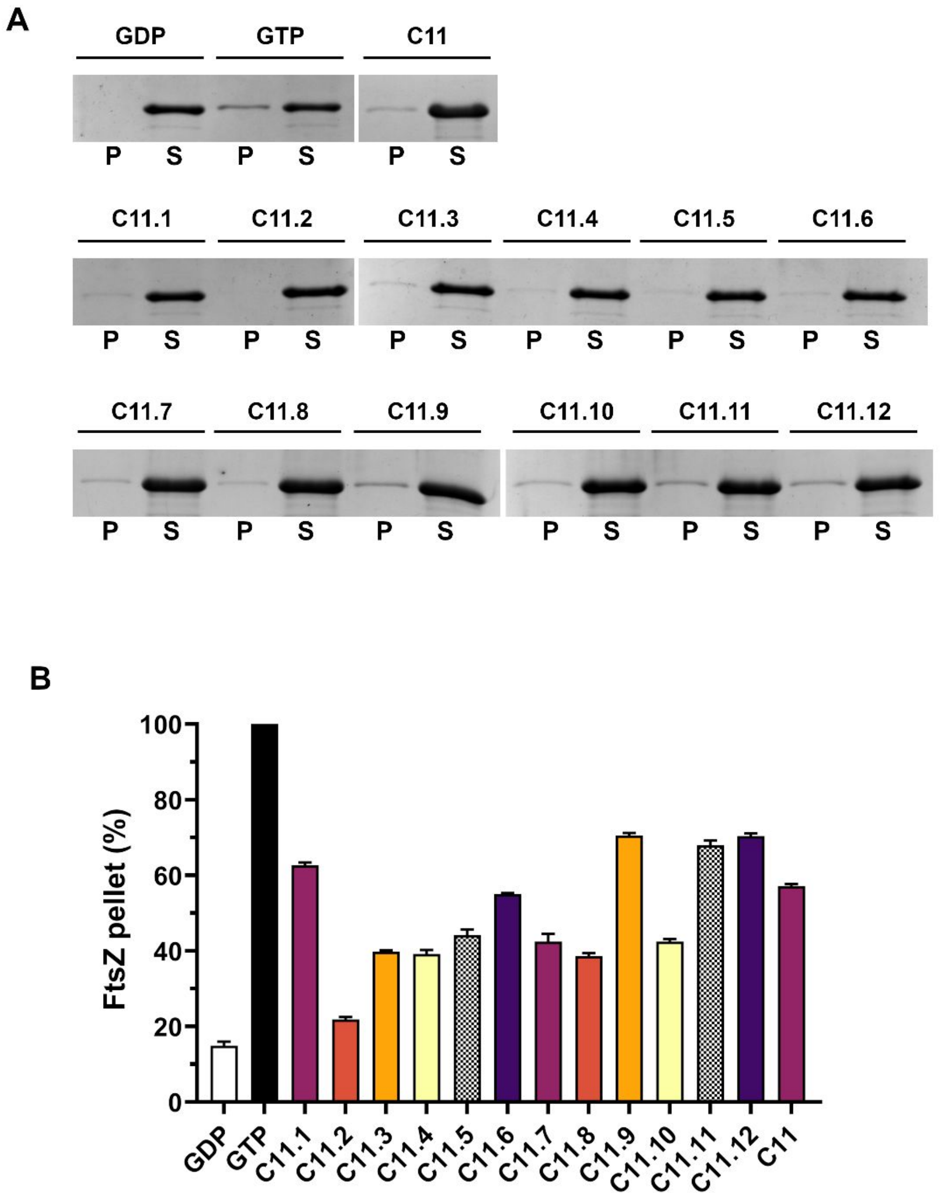

Figure S10. (**A**) FtsZSa sedimentation assay in the presence of the **C11** derivatives. GDP and GTP were used as negative and positive controls of the reaction, respectively. (P: pellet fraction; S: supernatant fraction). (**B**) Densitometric analysis of the pellet bands obtained from the sedimentation assay. The quantity of FtsZ in the pellet of the positive control (GTP) was set up as 100%. The densitometry analysis was carried out using ImageJ.

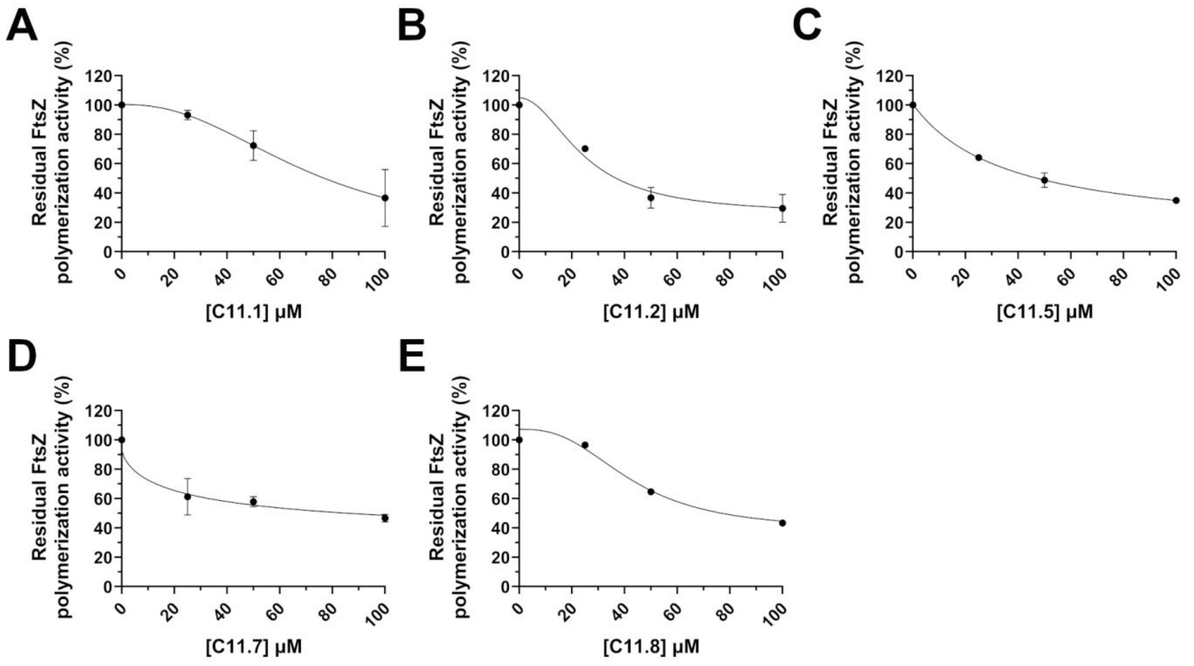

Figure S11. Dose-response curves of FtsZSa residual polymerization activity in the presence of **C11** analogues. (A) **C11.1**, (B) **C11.2**, (C) **C11.5**, (D) **C11.7** and (E) **11.8**.

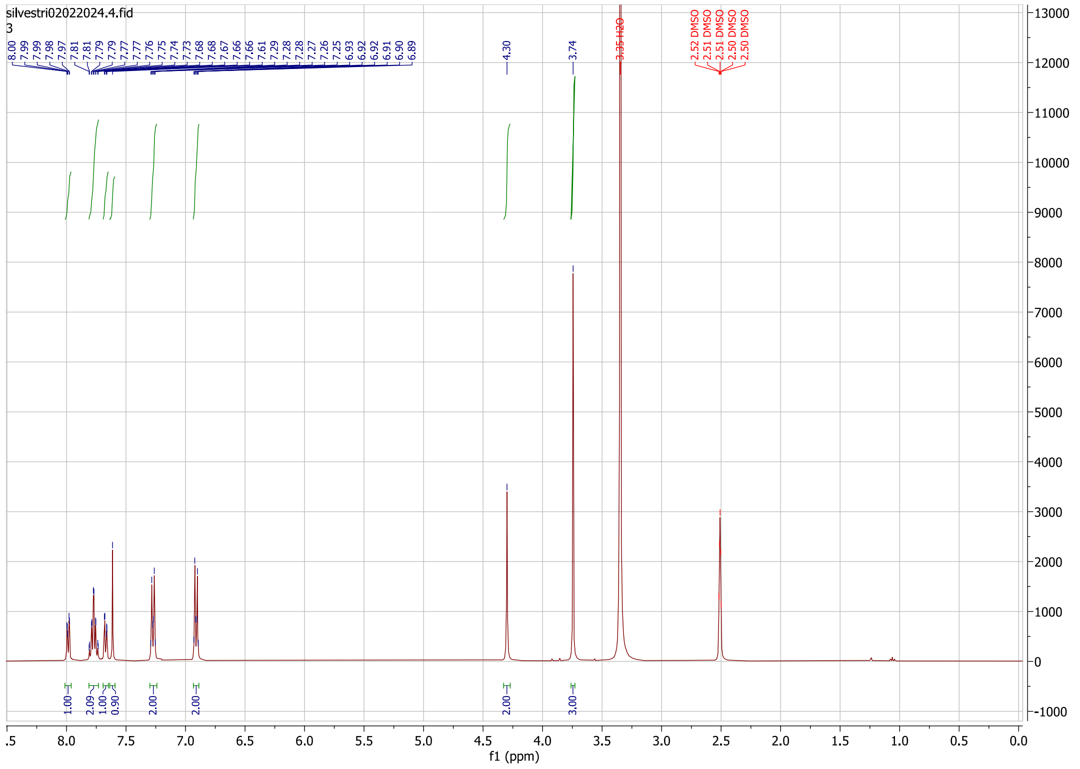

Figure S12 ^{1}H NMR spectrum of compound C11

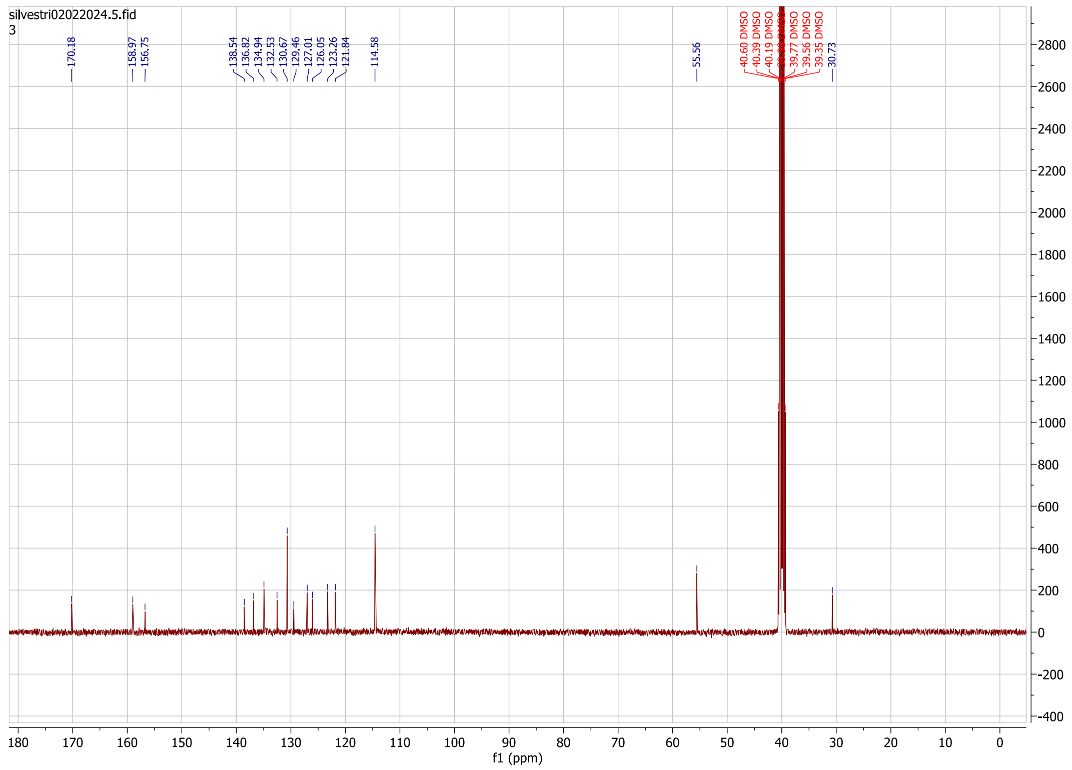

Figure S13 ^{13}C NMR spectrum of compound C11

Molecular Modelling

All the docking experiments were performed on a SuperMicro, Intel Xeon Silver powered machine (64 cores) running Ubuntu 20.04 LTS. The crystal structures of *S. aureus* FtsZ (PDB: 4DXD)[6] were downloaded from the Protein Data Bank (https://www.rcsb.org/) and prepared using Maestro (Schrödinger Release 2022-4: Maestro, Schrödinger, LLC, New York, NY, 2021).[7] Hydrogen atoms, missing side chains and missing loops were added while water and other solvents were removed. The 3D structures of the training set compounds were protonated at physiological pH and minimized using the OPLS4 force field included in Maestro suite.[8] Also drug-like properties were computed by QikProp.[9] Docking experiments were carried out by Glide.[10] Considering the crucial role played by the calcium atom in the allosteric pocket, it was introduced a constraint during the docking experiments to retain only molecules bearing moieties able to establish interaction with the cation. The pharmacophore model was developed by Phase.[11] It accounts for nine features: 1 H-bond acceptor; 2 H-bond donors; 3 hydrophobic points and 3 aromatic points. All the docking proposed conformations (10 per molecules) were filtered by the pharmacophore model and were retained only conformers that match 8 out of the 9 features. It was not fixed any required features. The images reported in this paper were generated by PyMol.[12]

In vitro FtsZ GTPase activity

FtsZSa and FtsZPa were expressed and purified as previously described.[13,14] GTPase activity was assayed at 30°C using a pyruvate kinase–L-lactic dehydrogenase (PK/LDH) spectrophotometric coupled assay, as previously described[15] with minor modification. The reaction mixture was first set up to contain 50 mM MES (pH 6.5), 5 mM $Mg(CH_3COO)_2$, 100 mM CH_3CO_2K, 10 U PK/LDH, 0.25 mM NADH, 0.25 mM phosphoenolpyruvate, and 4.8 µM of FtsZ. The assay was initiated by the addition of 1 mM GTP. The experiments were performed in triplicate. The inhibitory concentration that reduced the enzymatic activity by half (IC_{50}) was determined using Prism 10.

In vitro FtsZ polymerization Assay

The polymerization of FtsZ was assessed in vitro using a sedimentation protocol, as previously described.[15] The samples were analysed by SDS-PAGE on 12% polyacrylamide gels. The *in vitro* polymerization of the FtsZ protein was tested in the presence of **C11**. Light scattering assay of FtsZ polymerization was performed as previously described, by measuring the 90° angle light scattering with a Cary Eclipse Fluorescence Spectrophotometer (Varian) and using both excitation and emission wavelengths at 350 nm.[16] The polymerization was measured at 30°C, in 150 µL of 50 mM MES pH 6.5, 10 mM $Mg(CH_3COO)_2$, 100 mM CH_3CO_2K, using FtsZ at a final concentration of 12.5 µM, after addition of 1 mM GTP, and data were collected every 5 s.

Cryo-electron microscopy

Protein samples (FtsZSa) at the concentration of 12 µM in the presence or absence of inhibitor were shaked in a thermal block (300 rpm, 30°C, 2 min), then added with GTP/GDP 1 mM and shaked for further 5 min. A volume of 4 µl was immediately applied in a Vitrobot instrument (Thermo Fisher Scientific) to holey carbon grids (Quantifoil R 1.2/1.3 Cu 300/ C-Flat 1.2/1.3 Cu 300) that had been subjected in advance to glow discharge and blotted once for 3 to 5 seconds at 22°C and 95% relative humidity, then plunged into liquid ethane. Images were acquired by the software EPU (Thermo Fisher Scientific) on a 200kv Glacios cryo-transmission electron microscope (Thermo Fisher Scientific) equipped with a Falcon 3EC direct electron detector (Thermo Fisher Scientific) and were collected at 120000x magnification at a nominal defocus valute of -3.0 µM under low dose conditions.

Mammalian cell viability assay

Human adenocarcinomic alveolar basal epithelial cells A549 and human CF bronchial epithelial cells CFBE41o- (derived from a cystic fibrosis patient homozygous for the ΔF508 CFTR mutation) were maintained in routine culture with weekly splitting. Cells were cultured in Dulbecco's modified Eagle's medium (DMEM) supplemented with fetal bovine serum (10%), MEM non-essential amino acids (0.1 mM), penicillin (100 U/ml), streptomycin (100 µg/ml); CFBE41o- cells were cultured in minimal essential medium (MEM) supplemented with fetal bovine serum (10%), glutamine (2 mM), penicillin (100 U/ml), and streptomycin (100 µg/ml). Both cell cultures were maintained at 37°C in 5% CO_2 atmosphere. All reagents were purchased from Euroclone. Twenty-four hours before drug treatment, cells were trypsinized, resuspended in medium and

seeded to obtain a confluent monolayers in 96-wells plate. Different drug concentrations were tested and cell viability was assessed by 3-(4,5-dimethyl-2-thiazolyl)-2,5-diphenyl-2-H-tetrazolium bromide MTT assay at 3, 6 or 24 hours for each concentration. Specifically, MTT was added into wells at a final concentration of 0.5 mg/mL, incubated at 37°C for 2 hours to allow the formation of formazan, that was dissolved with DMSO. The formazan concentration was measured by reading OD560 with a Glomax discover (Promega) plate reader.[17] Cell viability data were derived from three independent experiments and were normalized to CT samples. CT samples were subjected only to DMSO solvent. Each assay was carried out in triplicates. Effect on mammalian tubulin was evaluated using the Tubulin Polymerization Assay, Fluorescence Based (Cytoskeleton, Inc.)

Bacterial strains and growth conditions.

Staphylococcus aureus ATCC25923, *S. aureus* clinical isolates (Table S1), *Pseudomonas aeruginosa* PAO1, *Acinetobacter baumannii* ATCC19606 and *Klebsiella pneumoniae* ATCC 13883 were grown aerobically in Mueller-Hinton-II (MH-II, Difco, BD) broth, if not differently specified, at 37°C at 200 rpm. *Escherichia coli* BL21DE3 strain (laboratory collection) was grown in LB broth at 37°C with shaking or on LB agar plates and used for recombinant protein expression. Kanamycin (PanReac, AppliChem) was used at 50 mg/L for plasmid selection and maintenance.

Minimum Inhibitory Concentration (MIC) Determination

The effectiveness of **C11** compound and of its derivatives against *S. aureus* ATCC25923, *S. aureus* clinical isolates, *P. aeruginosa* PAO1, *A. baumannii* ATCC19606 and *K. pneuomoniae* ATCC13883 was assessed determining MICs by the 2-fold microdilution method in U-bottom 96-well microtiter plates and inoculating about 10^5 CFU. The microtiter plates were incubated at 37°C for 24 h and growth was determined by the resazurin method.[18] A solution of resazurin sodium salt (Sigma Aldrich) was prepared at 0.01% in distilled water and filter-sterilized. Thirty microliters of resazurin solution were added to each well after 1 day of incubation at 37°C, and the microtiters were re-incubated at the same temperature for 3 h. The MIC was defined as the lowest concentration of the drug that prevented a change in colour from blue to pink, which indicates bacterial growth. Checkerboard assays of **C11** with concentrations ranging from 0 to 128 µg/ml in combination with the efflux pump inhibitor PaβN MC-207.110 (0–128 µg/ml) or Ceftazidime, Erythromycin, Meropenem,

Rifampicin or Tetracycline were set up, as previously described[19] and the results determined using the resazurin MIC method.

Time-Killing Curve

Time-killing curve of *S. aureus* ATCC25923 in the presence of C11 was performed as described by Silva et al.[20] Briefly, 0.5, 1 and 2 multiples of the MIC (2 μg/ml) were used. 20 ml of MH-II broth with the appropriate **C11** concentrations were inoculated with exponentially grown *S. aureus* ATCC25923 cells, to yield a final concentration of approximately 1×10^7 CFU/ml. The cultures were incubated at 37°C, and aliquots were removed at 0, 2, 4, 6, 8 and 24 hours for the determination of viable counts. Serial dilutions were spread on LB solid medium and the plates were incubated at 37°C O/N. Then, the number of colonies was determined. Killing curves were constructed by plotting the $\log_{10}$ CFU/ml vs. time. Bactericidal activity was defined as a reduction of 99.9% ($\geq 3 \log_{10}$) of the total number of CFU/ml in the original inoculum (NCCLS, 1999). Bacteriostatic activity was defined as maintenance of the original inoculum concentration or a reduction of less than 99.9% (<3 log10) of the total number of CFU/ml in the original inoculum.

Live cell imaging experiments

S. aureus ATCC25923 or *S. aureus* TD276[21-22] were cultured O/N in TSB, supplemented with Chloramphenicol 10 μg/ml in the case of strain TD276. Cells were diluted to OD of 0.02 in fresh TSB containing in the case of strain TD276 anhydrotetracycline 0.4 μM to induce the expression of mCherryFtsZ. After 1 hour of incubation at 37°C 2 μl of the culture was spotted onto an agar pad prepared with TSB and 1.5% agarose and with or without 0.4 μM anhydrotetracycline or 4 μg/ml of **C11**. For time-lapse microscopy experiments, an inverted widefield microscope (Leica DMi8S) equipped with a 100× oil immersion objective (HC PL FLUOTAR 100x/1.32 OIL) was used. The microscope was equipped with an environmental chamber maintained at 37°C. Images were recorded at 15-min intervals and up to 5 h. Bacteria were visualized in phase-contrast and fluorescence with the required parameters. Manual segmentation of individual cells and analysis of image stacks were performed using the ImageJ 1.52a software. The Selection Brush Tools was used to draw polygons corresponding to the shape of individual cells and to extract the cell planar area (μm^2). *S. aureus* TD276 cells were stained with Hoechst (1 μg/ml), were cultured on TSB agar pads as described and visualized in fluorescence (with the required parameters for Hoechst and mCherry) using a 160x objective (HC PL APO

160x/1.43 OIL, Leica). Images were deconvolved with Huygens essential version (Scientific Volume Imaging, The Netherlands, http://svi.nl).

Biofilm inhibition assay

The biofilm inhibitory activity of compound **C11** was tested on *S. aureus* ATCC25923 strain using the CFU counting method. The bacterial cells were cultured in TSB O/N at 37°C and diluted to 10^7–10^8 CFU/ml; then, 100 µl of culture were pipetted into the microtiter plate. After 2 hours of incubation, the supernatant (containing nonadherent cells) was removed and 100 µl of fresh sterile medium containing different concentrations of **C11** (2, 4 and 8 µg/ml) were added to each well and incubated for an additional 20 hours at 37°C. The formed biofilm was washed once with 200 µl of saline solution, and subsequently detached by scraping the bottom of the well with pipette tip resuspending cells in 100 µl of PS. The suspended biofilm was transferred to a new 96-well microplate followed by 10-fold dilutions prepared in PS. CFU were enumerated after 24 hours of incubation at 37°C. The experiment was performed three times with two technical replicates.

Biofilm Eradication Assay

To evaluate the biofilm eradication potential of **C11** on *S. aureus* ATCC25923, the protocol previously described[23] was used with minor modifications. Bacteria were cultured O/N in TSB and diluted to 1×10^7 CFU/ml in the same medium. Then, 100 µl of bacterial suspension were added to the wells of a flat-bottom 96-well microtiter plate and incubated for 2 hours at 37°C, allowing the bacterial adhesion to the abiotic surface. After that, wells were emptied and filled with 200 µl of fresh medium before incubating the microplate for an additional 20 hours at 37°C. The day after, the supernatants were removed, and the biofilms were washed with 100 µl of PS. TSB medium containing 4, 8 or 16 µg/ml of **C11** was added to the mature biofilms, and the plate was incubated for 24 hours at 37°C. Each condition was tested in triplicate, and untreated controls (TSB alone) were prepared. After 24 h, wells were rinsed two times with PS, completely removing planktonic bacteria, and the biofilms were disrupted by vigorous pipetting and scraping. The resulting microbial suspensions were serially diluted in saline solution and plated onto LB agar for the determination of the CFU/ml.

Biofilm Evaluation by Confocal Laser Scanning Microscopy

The biofilm inhibition was evaluated using confocal laser scanning microscopy as described below. Bacteria were cultured O/N in TSB and diluted to about 1 × 10^7 CFU/ml in the same medium. Bacterial suspension was added to the µ-Slide 4 Well Ibidi treated, incubated for two hours in TSB, at 37°C. After that the medium was removed and fresh TSB medium containing different concentrations of C11 (2, 4 and 8 µg/ml) was added. After an overnight incubation, the medium was removed, and biofilms were washed twice with saline solution and stained. Also biofilm eradication was evaluated using confocal laser scanning microscopy. Briefly, bacteria were cultured O/N in TSB and diluted to about 1 × 10^7 CFU/ml in the same medium. Bacterial suspension was added to the µ-Slide 4 Well Ibidi treated and incubated for two hours in TSB, at 37°C. Then, the medium was removed, and fresh TSB medium was added. After overnight incubation, the medium was removed and fresh TSB containing different concentrations of **C11** (4, 6 and 16 µg/ml) was added. After an overnight incubation, the medium was removed, and biofilms were washed twice with saline solution and stained. The staining of the biofilm was performed with Syto9 (Invitrogen) at a final concentration of 5 µM and in the case of biofilm eradication assays with both Syto9 and Propidium Iodide at the final concentration of 5 µM and 2.5 µM respectively. A SP8 point scanning confocal microscope (Leica, Germany) equipped with a 63× oil immersion objective (HC PL APO CS2 63x/1.40 OIL) and detection set to green fluorescence (representing Syto 9) and red fluorescence (representing Propidium Iodide) was used to take 3 snapshots randomly at different positions in the confocal field of each chamber. The Z-slices were obtained every 0.3 microns. For visualization and processing of biofilm images, ImageJ was used. The thickness, biomass, roughness coefficient, and biofilm distribution were measured using the COMSTAT 2 software.[24] All confocal scanning laser microscopy experiments were performed three times, and standard deviations were measured.

Evaluation of C11 efficacy in the *Galleria mellonella* infection model

Larvae were purchased from a local provider in Pavia and grouped in petri dishes (at least 10 larvae/group) according to their weight. The experiment was carried out as previously described with minor modification.[25] Inoculation with a lethal dose (10^5 CFU) of mid-exponential phase *S. aureus* ATCC25923, or saline solution (control), was carried out with an injection volume of 10 µl. After 2 hours of incubation at 30°C in the dark, mock and *S. aureus* infected larvae were administered with 10 µl of C11 (40 mg/kg), DMSO (8%) and re-incubated in the same conditions for 3 days. Larval viability was registered after 24, 48, 72 hours and 6 days considering the lack of movement after tactile stimulus, suggestive of larval death.

Chemistry

The compound **C11** structure was checked by, 1H and C13 NMR analyses. Proton nuclear magnetic resonance (^{1}H NMR) spectra were recorded with a Bruker Avance (400 MHz) spectrometer in the indicated solvent, and the corresponding fid files were processed with MestreLab Research SL MestreReNova 6.2.1–769 software. Carbon-13 nuclear magnetic resonance (^{13}C NMR) spectra were recorded with a Bruker AVANCE (100 MHz) spectrometer in the indicated solvent, and the corresponding FID files were processed by MestreLab Research SL MestreReNova 6.2.1–769 software. Chemical shifts of ^{1}H and ^{13}C NMR are expressed in δ units (ppm) from tetramethylsilane. The Spectrums were reported at the supplementary materials (Figure S12 and S13). Melting point (mp) was determined on a Stuart Scientific SMP1 apparatus and is uncorrected.

Compound **C11**: *3-((5-(4-methoxybenzyl)-1,3,4-oxadiazol-2-yl)thio)benzo[b]thiophene 1,1-dioxide*. Mp 190-191 °C.^{1}H NMR (DMSO-d_6, 400 MHz): δ 3.74 (s, 3H), 4.30 (s, 2H), 6.92 (d, J = 8.0 Hz, 2H), 7.28 (d, J = 8.0 Hz, 2H), 7.61 (S, 1H), 7.66-7.68 (m, 1H), 7.73-7.81 (m, 2H) and 7.97-8.00 (m, 1H). ^{13} C NMR (DMSO-d_6, 100 MHz): δ 30.73, 55.56, 114.58, 121.84, 123.26, 126.05, 127.01, 129.46, 130.67, 132.53, 134.94, 136.82, 138.54, 156.75, 158.97 and 170.18 ppm.

Compounds Purity

Compound **C11** and all the close analogue reported in Table 3 (**C11.1** to **C11.12**) were purchased by Specs.[3] The purity for all the compounds was declared by vendors higher than 95%.

Reference

1. Hogan AM, Scoffone VC, Makarov V, Gislason AS, Tesfu H, Stietz MS, Brassinga AKC, Domaratzki M, Li X, Azzalin A, et al. Competitive Fitness of Essential Gene Knockdowns Reveals a Broad-Spectrum Antibacterial Inhibitor of the Cell Division Protein FtsZ. Antimicrob Agents Chemother. 2018; 62(12):e01231-18.

2. Lifechemicals. https://lifechemicals.com

3. Specs. https://www.specs.net

4. Do T, Schaefer K, Santiago AG, Coe KA, Fernandes PB, Kahne D, Pinho MG, Walker S. Staphylococcus aureus cell growth and division are regulated by an amidase that trims peptides from uncrosslinked peptidoglycan. Nat Microbiol. 2020; 5(2):291-303.

5. Visca P, Pisa F, Imperi F. The antimetabolite 3-bromopyruvate selectively inhibits Staphylococcus aureus. Int J Antimicrob Agents. 2019; 53(4):449-455.

6. Tan CM, Therien AG, Lu J, Lee SH, Caron A, Gill CJ, Lebeau-Jacob C, Benton-Perdomo L, Monteiro JM, Pereira PM, et al. Restoring methicillin-resistant *Staphylococcus aureus* susceptibility to β-lactam antibiotics. Sci Transl Med. 2012; 4(126):126ra35.

7. Sastry GM, Adzhigirey M, Day T, Annabhimoju R, Sherman W. Protein and ligand preparation: parameters, protocols, and influence on virtual screening enrichments. J Comput Aided Mol Des. 2013; 27(3):221-34.

8. Lu C, Wu C, Ghoreishi D, Chen W, Wang L, Damm W, Ross GA, Dahlgren MK, Russell E, Von Bargen CD, et al. OPLS4: Improving Force Field Accuracy on Challenging Regimes of Chemical Space. J Chem Theory Comput. 2021; 17(7):4291-4300.

9. Schrödinger Release 2023-1: QikProp, Schrödinger, LLC, New York, NY, 2023

10. Yang Y, Yao K, Repasky MP, Leswing K, Abel R, Shoichet BK, Jerome SV. Efficient Exploration of Chemical Space with Docking and Deep Learning. J Chem Theory Comput. 2021; 17(11):7106-7119.

11. Dixon SL, Smondyrev AM, Knoll EH, Rao SN, Shaw DE, Friesner RA. PHASE: a new engine for pharmacophore perception, 3D QSAR model development, and 3D database screening: 1. Methodology and preliminary results. J Comput Aided Mol Des. 2006; 20(10-11):647-71.

12. The PyMOL Molecular Graphics System, Version 2.0 Schrödinger, LLC.

13. Trespidi G, Scoffone VC, Barbieri G, Marchesini F, Abualsha'ar A, Coenye T, Ungaro F, Makarov V, Migliavacca R, De Rossi E, Buroni S. Antistaphylococcal Activity of the FtsZ Inhibitor C109. Pathogens. 2021; 10(7):886.

14. Chiarelli LR, Scoffone VC, Trespidi G, Barbieri G, Riabova O, Monakhova N, Porta A, Manina G, Riccardi G, Makarov V, Buroni S. Chemical, Metabolic, and Cellular Characterization of a FtsZ Inhibitor Effective Against *Burkholderia cenocepacia*. Front Microbiol. 2020; 7(11):562.

15. Hogan AM, Scoffone VC, Makarov V, Gislason AS, Tesfu H, Stietz MS, Brassinga AKC, Domaratzki M, Li X, Azzalin A, et al. Competitive Fitness of Essential Gene Knockdowns Reveals a Broad-Spectrum Antibacterial Inhibitor of the Cell Division Protein FtsZ. Antimicrob Agents Chemother. 2018; 62:e01231-18.

16. Król E, Scheffers DJ. FtsZ polymerization assays: simple protocols and considerations. J Vis Exp. 2013; 16(81): e50844.

17. Zheng YY, Du RL, Cai SY, Liu ZH, Fang ZY, Liu T, So LY, Lu YJ, Sun N, Wong KY. Study of Benzofuroquinolinium Derivatives as a New Class of Potent Antibacterial Agent and the Mode of Inhibition Targeting FtsZ. Front Microbiol. 2018; 17(9):1937.

18. Mann CM, Markham JL. A new method for determining the minimum inhibitory concentration of essential oils. J Appl Microbiol. 1998; 84(4):538-44.

19. Saiman L, Mehar F, Niu WW, Neu HC, Shaw KJ, Miller G, Prince A. Antibiotic susceptibility of multiply resistant *Pseudomonas aeruginosa* isolated from patients with cystic fibrosis, including candidates for transplantation. Clin Infect Dis. 1996; 23(3):532-7.

20. Silva F, Lourenço O, Queiroz JA, Domingues FC. Bacteriostatic versus bactericidal activity of ciprofloxacin in Escherichia coli assessed by flow cytometry using a novel far-red dye. J Antibiot (Tokyo). 2011; 64(4):321-5.

21. Monteiro JM, Pereira AR, Reichmann NT, Saraiva BM, Fernandes PB, Veiga H, Tavares AC, Santos M, Ferreira MT, Macário V, et al. Peptidoglycan synthesis drives an FtsZ-treadmilling-independent step of cytokinesis. Nature. 2018; 554(7693):528-532.

22. Do T, Schaefer K, Santiago AG, Coe KA, Fernandes PB, Kahne D, Pinho MG, Walker S. *Staphylococcus aureus* cell growth and division are regulated by an amidase that trims peptides from uncrosslinked peptidoglycan. Nat Microbiol. 2020; 5(2):291-303.

23. She P, Luo Z, Chen L, Wu Y. Efficacy of levofloxacin against biofilms of *Pseudomonas aeruginosa* isolated from patients with respiratory tract infections in vitro. Microbiologyopen. 2019; 8(5):e00720.

24. Heydorn A, Nielsen AT, Hentzer M, Sternberg C, Givskov M, Ersbøll BK, Molin S. Quantification of biofilm structures by the novel computer program COMSTAT. Microbiology (Reading). 2000; 146:2395-2407.

25. Silva LN, Da Hora GCA, Soares TA, Bojer MS, Ingmer H, Macedo AJ, Trentin DS. Myricetin protects *Galleria mellonella* against *Staphylococcus aureus* infection and inhibits multiple virulence factors. Sci Rep. 2017; 7(1):2823.
